# Unraveling the genetics of heat tolerance in chickpea landraces (*Cicer arietinum* L.) using genome-wide association studies

**DOI:** 10.3389/fpls.2024.1376381

**Published:** 2024-03-25

**Authors:** Thippeswamy Danakumara, Neeraj Kumar, Basavanagouda Siddanagouda Patil, Tapan Kumar, Chellapilla Bharadwaj, Pradeep Kumar Jain, Manduparambil Subramanian Nimmy, Nilesh Joshi, Swarup Kumar Parida, Shayla Bindra, Chittaranjan Kole, Rajeev K. Varshney

**Affiliations:** ^1^ ICAR- Indian Agricultural Research Institute, New Delhi, India; ^2^ International Centre for Agricultural Research in the Dry Areas, Amlaha, Madhya Pradesh, India; ^3^ ICAR-National Institute for Plant Biotechnology, New Delhi, India; ^4^ National Institute of Plant Genome Research (NIPGR), New Delhi, India; ^5^ Punjab Agricultural University, Ludhiana, India; ^6^ Prof. Chittaranjan Kole Foundation for Science & Society, Kolkatta, India; ^7^ Murdoch University, Murdoch, WA, Australia

**Keywords:** genome-wide association study (GWAS), chickpea, heat, single-nucleotide polymorphism (SNPs), QTN

## Abstract

Chickpea, being an important grain legume crop, is often confronted with the adverse effects of high temperatures at the reproductive stage of crop growth, drastically affecting yield and overall productivity. The current study deals with an extensive evaluation of chickpea genotypes, focusing on the traits associated with yield and their response to heat stress. Notably, we observed significant variations for these traits under both normal and high-temperature conditions, forming a robust basis for genetic research and breeding initiatives. Furthermore, the study revealed that yield-related traits exhibited high heritability, suggesting their potential suitability for marker-assisted selection. We carried out single-nucleotide polymorphism (SNP) genotyping using the genotyping-by-sequencing (GBS) method for a genome-wide association study (GWAS). Overall, 27 marker–trait associations (MTAs) linked to yield-related traits, among which we identified five common MTAs displaying pleiotropic effects after applying a stringent Bonferroni-corrected p-value threshold of <0.05 [−log_10_(p) > 4.95] using the BLINK (Bayesian-information and linkage-disequilibrium iteratively nested keyway) model. Through an in-depth *in silico* analysis of these markers against the CDC Frontier v1 reference genome, we discovered that the majority of the SNPs were located at or in proximity to gene-coding regions. We further explored candidate genes situated near these MTAs, shedding light on the molecular mechanisms governing heat stress tolerance and yield enhancement in chickpeas such as indole-3-acetic acid–amido synthetase GH3.1 with GH3 auxin-responsive promoter and pentatricopeptide repeat-containing protein, etc. The harvest index (HI) trait was associated with marker Ca3:37444451 encoding aspartic proteinase ortholog sequence of *Oryza sativa* subsp. *japonica* and *Medicago truncatula*, which is known for contributing to heat stress tolerance. These identified MTAs and associated candidate genes may serve as valuable assets for breeding programs dedicated to tailoring chickpea varieties resilient to heat stress and climate change.

## Introduction

Chickpea (*Cicer arietinum* L.) is an important annual legume plant with a genome size of 738 Mb (Varshney et al., 2013). India stands as the world’s largest producer of chickpeas, contributing to 75% of the global production (Gaur et al., 2019). Recent advancements in genetic resource enhancement for chickpeas involve next-generation sequencing (NGS) initiatives, which have revolutionized genomics research. NGS has enabled cost-effective and rapid sequencing of chickpea genomes, unveiling thousands of genetic markers that hold immense potential for enhancing chickpea breeding (Varshney et al., 2019). Additionally, genomics research plays a pivotal role in identifying the specific genes governing vital traits in chickpeas, including drought, salinity, and heat tolerance (Bhat et al., 2020; Jha et al., 2021; Kumar et al., 2021).

The ongoing impact of climate change has brought significant changes in Indian agriculture, resulting in a shift toward chickpea cultivation in warmer regions (Mannur et al., 2019). This change has exposed chickpea crops to drought and heat stress, leading to yield penalties of up to 70%. In India, an increase in seasonal temperature of 1°C leads to a yield reduction of 474 kg/ha, and high temperatures (≥35°C) can lead to a 39% overall yield reduction in chickpeas (Jain et al., 2023). Also, as per the intergovernmental panel on climate change, the current rate of global warming is 0.2°C per decade and is predicted to touch 1.5°C between 2030 and 2050 (Mohanty et al., 2024). Such rising temperatures will lead to high heat stress and a severe threat to global food security by impacting yield losses of up to 10% to 15% in most of the food crops for every 1°C rise in the optimum temperature (Devasirvatham and Tan, 2018).

The intricate nature of heat stress tolerance, which involves multiple genes with pleiotropic and epistatic effects, presents a substantial challenge in breeding. Phenotypic evaluation in earlier studies has identified some heat-tolerant genotypes in chickpeas (Jha et al., 2022). However, there are no reports of screening chickpea landraces and wild germplasm found near the region of crop domestication [West Asia and North Africa (WANA) region] for heat stress tolerance. Most research on chickpeas to date has been centered on varietal evaluation for yield and growth traits at a specific developmental stage for heat stress tolerance. However, the expanding genomic resources have facilitated the identification of heat stress-responsive quantitative trait loci (QTLs) in chickpeas through QTL mapping and genome-wide association studies, using both mapping populations and natural germplasm collections (Kushwah et al., 2021). However, only a limited number of initiatives have successfully identified specific genes/alleles or significant QTLs that confer heat tolerance across a wide range of genetic backgrounds. Given the critical importance of developing heat stress-tolerant varieties in today’s context, the pursuit to identify potential and major genomic loci that regulate heat tolerance using the landraces can be a valuable strategy for heat stress breeding programs.

With this background and aim, the current study focused on the identification of QTLs for heat tolerance in 153 chickpea landraces. These landraces were selected to encompass a broad spectrum of genetic diversity and geographical origins. Our investigation focused on an in-depth exploration of growth traits, spanning from the flowering stage to harvestable maturity, employing robust phenotyping methodologies. Genome-wide association studies (GWASs) were conducted to delve into the interrelationships among various agronomic traits to identify the potential marker–trait association for heat stress tolerance.

## Materials and methods

### Plant material and experimental setup

The association panel under study comprised 153 diverse landraces along with one tolerant check variety JG14 in the current investigation, predominantly representing the centers of diversity for chickpeas in the WANA region. These landraces were procured from the chickpea molecular breeding laboratory, Division of Genetics, ICAR-Indian Agricultural Research Institute, New Delhi (**Supplementary Table 1**). Multi-environment trials (METs) were conducted at three distinct geographic locations, i.e., Amlaha, Dharwad, and Delhi, for the two consecutive years, 2021–22 and 2022–23 (**Table 1**). The trials encompassed two growing conditions, timely sown and late sown (30 days after the former). The experiment was meticulously designed using an alpha lattice design. Each genotype was replicated three times at each location. All the genotypes were randomly distributed and sown in plots with rows measuring 2 m in length, a plant spacing of 30 cm, inter-row spacing of 50 cm, inter-plot spacing of 1.5 meters, and a 2.0-m gap between replications. All the standard recommended package of practices was followed to raise a good chickpea crop in each location.

**Table 1:** Details of sowing conditions, locations, and year of experiment along with abbreviations.

Treatment	Location	Code	Latitude	Longitude	Altitude	Year	Abbreviation
Amlaha_Normal	International Center for Agriculture Research in the Dry Areas (ICARDA), Amlaha, Madhya Pradesh, India	E1	23.14711	76.92035	502 m	2021	AN_2021
Amlaha_Late	International Center for Agriculture Research in the Dry Areas (ICARDA), Amlaha, Madhya Pradesh, India	E2	23.14711	76.92035	502 m	2021	AL_2021
Dharwad_Normal	ICAR-IARI-Regional Research Station, Dharwad, Karnataka, India	E3	15.45102	75.00844	678 m	2021	DN_2021
Dharwad_Late	ICAR-IARI-Regional Research Station, Dharwad, Karnataka, India	E4	15.45102	75.00844	678 m	2021	DL_2021
Delhi_Normal	ICAR-Indian Agricultural Research Institute (IARI)-New Delhi, India	E5	28.08	77.12	228.61 m	2021	DeN_2021
Delhi_Late	ICAR-Indian Agricultural Research Institute (IARI)-New Delhi, India	E6	28.08	77.12	228.61 m	2021	DeL_2021
Amlaha_Normal	International Center for Agriculture Research in the Dry Areas (ICARDA), Amlaha, Madhya Pradesh, India	E7	23.14711	76.92035	502 m	2022	AN_2022
Amlaha_Late	International Center for Agriculture Research in the Dry Areas (ICARDA), Amlaha, Madhya Pradesh, India	E8	23.14711	76.92035	502 m	2022	AL_2022
Dharwad_Normal	ICAR-IARI-Regional Research Station, Dharwad, Karnataka, India	E9	15.45102	75.00844	678 m	2022	DN_2022
Dharwad_Late	ICAR-IARI-Regional Research Station, Dharwad, Karnataka, India	E10	15.45102	75.00844	678 m	2022	DL_2022
Delhi_Normal	ICAR-Indian Agricultural Research Institute (IARI)-New Delhi, India	E11	28.08	77.12	228.61 m	2022	DeN_2022
Delhi_Late	ICAR-Indian Agricultural Research Institute (IARI)-New Delhi, India	E12	28.08	77.12	228.61 m	2022	DeL_2022

### Phenotypic observations

The chickpea landraces were extensively phenotyped at all the locations for both the conditions for all the two years for recording the following quantitative traits: days to 50% flowering (DTF; number of days), days to maturity (DTM; number of days), plant height (PH; cm), biomass yield (BY; g/1 m of row), hundred-seed weight (HSW; g), plot yield (g/1 m of row), and harvest index (HI), which was calculated by dividing plot yield by biomass yield.

### SNP genotyping

For high-throughput genotyping, DNA was isolated from leaves of 7-day-old seedlings grown under controlled conditions. The DNA extraction procedure followed the cetyl trimethylammonium bromide (CTAB) method originally described by Murray and Thompson in 1980 and modified by Kumar et al. (2013). To assess DNA quality, 0.8% agarose gel electrophoresis was used, and DNA samples measuring 30 ng/µL were selected for subsequent single-nucleotide polymorphism (SNP) genotyping. FASTQ files of all the genotypes having 64-bp sequences with barcode adopters and common adopters were sorted, and counted sequence tags were merged to TagCount files used to align to Reference genome CDC Frontier v1 and were used for TagsOnPhysicalMap (TOPM). Parallelly, Master TagCounts + original FASTAQ files determined the distribution of master tags among samples (taxa) to generate the TagsByTaxa (TBT) file. The combined TagsOnPhysicalMap (TOPM) + TagsByTaxa (TBT) file was used for calling and filtering SNP and updating TOPM with variants followed by the production of a ready TOPM file. Finally, the HapMap Format (hmp) was provided and used for the GWAS analysis after filtering and imputation. Genotyping by sequencing (GBS) was employed to obtain SNP marker data in the HapMap format, which comprised 16,892 SNPs. Subsequently, after filtering for monomorphic alleles and considering criteria such as a minor allele frequency (MAF) of less than 0.05, a missing data frequency exceeding 0.18, and a heterozygote frequency surpassing 0.20, a total of 4,530 SNPs were retained for the subsequent GWAS analysis using TASSEL 5.0 (Bradbury et al., 2007).

### Data analysis

The phenotypic data generated in multi-location trials (MLTs) under different conditions were analyzed using the R package agricolae version 1.3-6. The comprehensive analysis includes ANOVA and adjusted means for each genotype based on the alpha lattice design. Principal component analysis (PCA) was carried out using the R package FactoMineR version 2.4. The graphical representation of the PCA results was generated using the R package factoextra version 1.0.7 (Kassambara, 2020). Furthermore, Pearson’s correlation coefficients were computed to assess the relationships among the studied traits, and visual representations of these correlations were constructed using the R package corrplot (Wei et al., 2017).

For effective analysis of genotypic data, a total of 4,530 SNPs, evenly distributed at approximately 1-Mb intervals across the genome, were filtered and utilized to assess the population structure using the STRUCTURE version 2.3.4 (Pritchard et al., 2010). The analysis was conducted with the following specific parameters: 100,000 burn-in cycles and 100,000 Markov chain Monte Carlo (MCMC) with three iterations performed for each k value ranging from 1 to 10. The optimal number of subpopulations (delta K) was determined using the Evanno (Evanno et al., 2005) method outlined by Structure Harvester (http://taylor0.biology.ucla.edu/structureHarvester/). In the context of cluster analysis, a distance matrix was generated using TASSEL version 5. Subsequently, a neighbor-joining (NJ) tree file in Newick format was exported to iTOL version 6.5.2 (accessible at https://itol.embl.de/). This allowed us to create a dendrogram using the neighbor-joining method. PCA and kinship analysis based on SNP markers were carried out using the GAPIT (Lipka et al., 2012). Graphical representation of the genetic position of MTA-QTLs was carried out using MapChart 2.3 (https://www.wur.nl/en/show/mapchart.htm).

### Association mapping analysis

We computed the r^2^ values for 4,530 pairs of SNP markers, then filtered them focusing on pairs within each chromosome, and created a linkage disequilibrium heat map to identify significant linkage disequilibrium (LD) block and its size, which falls diagonally in the heat map at a p-value of 0.001. We generated a whole genome and sorted for individual chromosomes by utilizing TASSEL version 5. Subsequently, we used these files to generate LD decay curves for all eight chromosomes individually and for the entire genome. To estimate the sizes of LD blocks, we plotted the r^2^ values against the distance in base pairs (bp) while setting a threshold at r^2 =^ 0.2 and recorded the distance at which LD decay reached its midpoint. For the genome-wide association analysis, we utilized the 4,530 SNP markers along with the adjusted mean and best linear unbiased prediction (BLUP) values obtained from META-R (Multi Environment Trial Analysis with R for Windows) version 6.0 in linear mixed models. These models estimated random effects for each trait, and we analyzed them using GAPIT version 3 in R with PCA 3 as a default parameter (Wang & Zhang, 2021). We chose the BLINK (Bayesian-information and linkage-disequilibrium iteratively nested keyway) model for its computational efficiency and ability to avoid false positives in identifying quantitative trait nucleotides (QTNs) (Huang et al., 2019; Zhou et al., 2023), as it considers computationally efficient fixed effect model (FEM) and avoids computationally expensive random effect model (REM). To evaluate the quality of the association model fitting, we employed a quantile–quantile (Q–Q) plot, which compared the expected and observed −log_10_(p) values. To ensure stringency in our selection of marker–trait associations (MTAs), we applied the Bonferroni correction. This entailed establishing a significance threshold at −log_10_(p) value of 0.05 divided by the total number of markers, which was 4,530, in order to minimize the risk of false positives. We visualized these significant MTAs using a Manhattan plot. We also identified stable MTAs across different locations. Moreover, we pinpointed pleiotropic SNPs, which were associated with multiple traits simultaneously. To further explore the potential candidate genes linked to these significant SNPs, we conducted a search for gene-coding regions within a 100-kb flanking region of the MTAs. We performed this search against the NCBI Reference genome ASM33114v1, which has a size of 530.8 Mb. We utilized the pulse database for chickpea (https://www.pulsedb.org/blast/report/) for annotating these significant QTNs.

## Results

### Phenotypic variability in chickpea landraces

**Figure 1:**
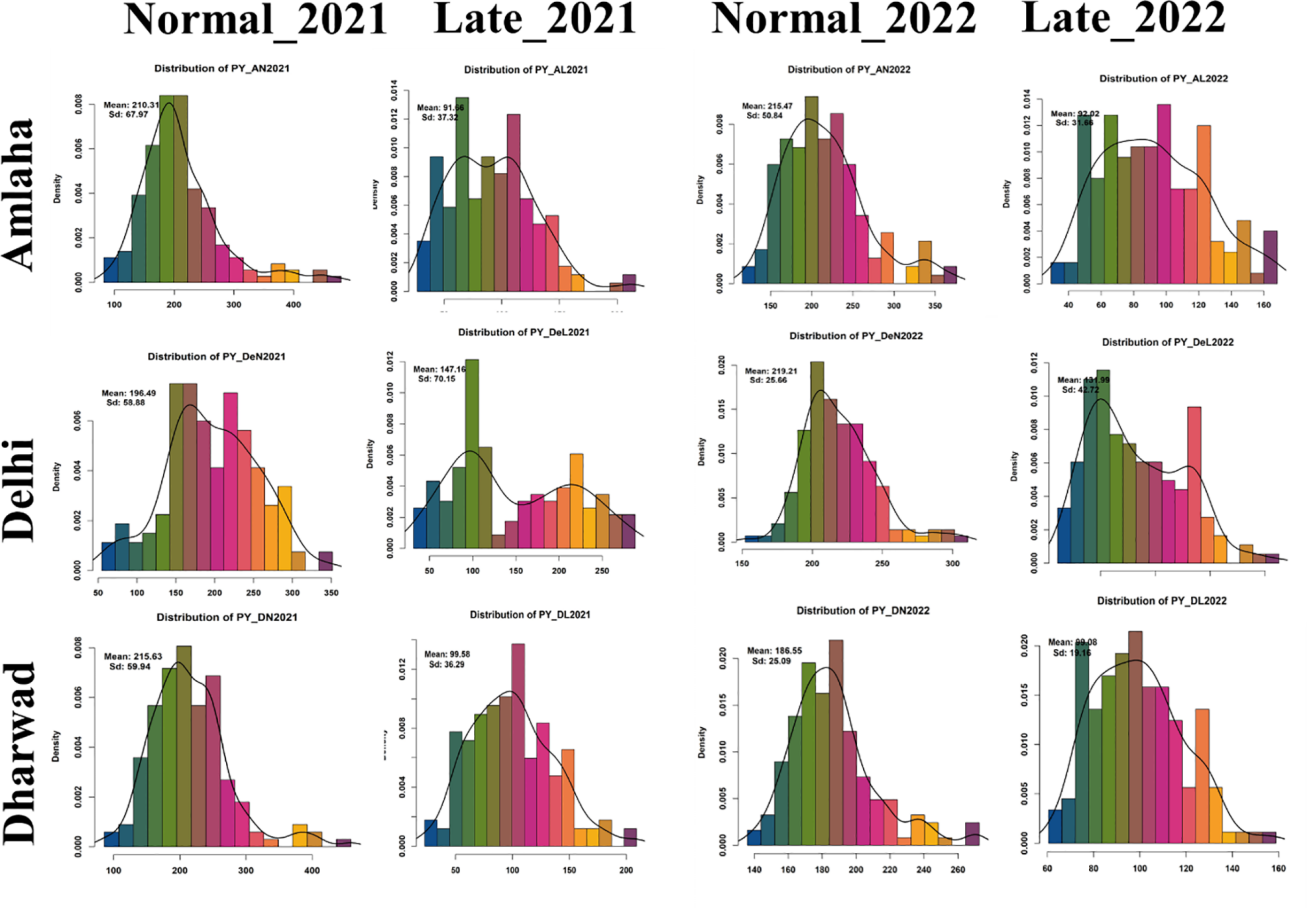
Frequency distribution plot yield (g) (PY) conducted under timely and late conditions in consecutive years 2021 and 2022.

The pooled analysis of variance indicated a significant difference between the studied traits for genotype, treatment, season, location, genotype by treatment, and treatment by location. Furthermore, we noticed the genotype by treatment by location was non-significant for DTF, DTM, and HSW (**Table 2**). The frequency distribution curve indicated the normal to near-normal distribution for the majority of the traits under normal timely sown and late-sown conditions (**Figure 1**; **Supplementary Figure 1**). We performed the Shapiro–Wilk test to prove this statement. p-Value ≥0.05 is considered a normal distribution, ≥0.001 is a near-normal distribution, and ≤0.001 is a non-normal distribution.

**Table 2:** ANOVA mean sum of squares of each trait tested under study.

Source of variation	Df	DTF	DTM	PH	HSW	BY	HI	PY
GENOTYPE	153	708***	356***	738***	1,003***	543,063***	769***	67,316***
TREATMENT	1	885,733***	1,556,913***	160,880***	9,052***	73,241,193***	60,447***	12,960,302***
SEASON	1	1,728***	3,790***	1***	302***	1,179,669***	4,923***	16,944*
REP	2	8,171***	3,958***	491***	1,534***	1,574,596***	6,820***	193,777***
LOCATION	2	38,389***	17,503***	30,586***	105,305***	1,210,706***	4,167***	287,329***
REP : BLOCK	12	503***	95 ns	190***	138***	128,607***	59 ns	22,256***
GENOTYPE : LOCATION	306	113***	59 ns	156***	121***	115,563***	209***	19,136***
GENOTYPE : TREATMENT	153	285***	151***	381***	91***	288,050***	452***	23,262***
TREATMENT : LOCATION	2	2,176***	64,570***	45,287***	367***	800,084***	7,496***	325,546***
GENOTYPE : TREATMENT : LOCATION	306	72 ns	39 ns	94***	18 ns	53,085***	100***	5,557***
Residuals	4,605	70	59	57	45	30,552	69	4,261
LSD		3.86	3.53	1.686352	3.08	80.76	3.82	30.16

ns, non-significant; Df, degrees of freedom; DTF, days to flowering (days); DTM, days to maturity (days); PH, plant height (cm); HSW, hundred-seed weight (g); BY, biological yield (g); HI, harvest index (%); PY, plot yield (g).

Significance of the difference between landraces at *p < 0.05; ***p < 0.001.

The mean values for the traits exhibited significant variations across locations. Under normal sown conditions across locations, environmental variation ranges from 4.73 for DTM_DN2021 to 82,184.86 for the (BY_DeN2022) trait. The genotypic variation ranges from 3.05 for DTM_DeN2021 to 76,340.1 for the BY_DN2021 trait. While phenotypic variation ranges from 18.01 for HSW_DeN2021 to 140,100.4 for BY_AN2022. The broad-sense heritability ranges from 7.75% (HI_DeN2022) to 95% (DTM_DN2021), and genetic advance ranges from 1.21 (DTM_DeN2021) to 501.94 (BY_DN2021). Notably, under late-sown conditions, we observed the highest phenotypic variation for traits such as biological yield, plot yield, and harvest index. This was coupled with broad-sense heritability figures ranging from 13.24% (for BY_DL2022) to 93% (for DTM_DL2021). We observed that the genotypic mean square for HI_DeN2022 was non-significant, but all the remaining traits show significant differences at p < 0.001 (**Supplementary Table 2**).

### Phenotypic correlation and PCA

Pearson’s correlation coefficient calculated for the traits for both conditions indicated a positive correlation among the traits such as plot yield (PY), BY, and HI across various locations. However, PY showed a negative correlation with DTM (p < 0.05) and exhibited non-significant correlations with PH, DTF, and HSW under normal sown conditions across different locations. In the late-sown condition, we observed negative correlations between DTF and DTM with PY, while PY showed non-significant correlations with HSW (**Supplementary Figure 2**). The PCA under the normal sown conditions of Amlaha in 2021 showed that the first principal component explained 27.54% of the variation. The primary contributors to this component were BY and PY. Meanwhile, the second dimension, which accounted for 24.7% of the variation, was influenced by traits like PH and DTF, with additional contributions from HSW and HI. In the third dimension, HSW and HI played significant roles, contributing to the variation. DTM had an impact on the fifth dimension. Notably, BY and PY were closely clustered together in the analysis, forming an acute angle, which indicated a positive correlation between these two traits. However, HSW, DTF, DTM, and PH clustered together at an acute angle but had a straight angle with HI, suggesting a negative correlation between HI and HSW, DTF, DTM, and PH traits. Similarly, we observed that DTM and DTF were negatively correlated to HI and PY where an angle of 180° between coordinate lines contributed to PC1 of 39.6%, similar to BY and HSW under the late-sown condition of Amlaha 2021. PCA in Amlaha 2022 indicated that dimension 1 explains 26.6% and 38.1% with dimension 2 explaining 21.8% and 21.1% for normal and late-sown conditions, respectively. In Delhi 2021, under controlled conditions, dimension 1 accounted for 37.9% of the variance, and dimension 2 for 21.3%. For late conditions, dimension 1 explained 34.9%, and dimension 2 explained 17.9%. In Delhi 2022, for timely sowing, dimension 1 explained 29.5%, while dimension 2 explained 23.8%. For late sowing, dimension 1 explained 36.9%, and dimension 2 explained 18.8%. For Dharwad 2021, under controlled conditions, dimension 1 contributed to 27.2% of the variance and dimension 2 to 24.8%. For late conditions, dimension 1 explained 36.5%, and dimension 2 explained 22.4%. In Dharwad 2022, for timely sowing, dimension 1 accounted for 29.9%, and dimension 2 for 21.9% of the variance. For late sowing, dimension 1 explained 37.4%, and dimension 2 explained 19.2% (**Supplementary Figure 3**).

### Genome-wide SNP marker distribution

**Figure 2:**
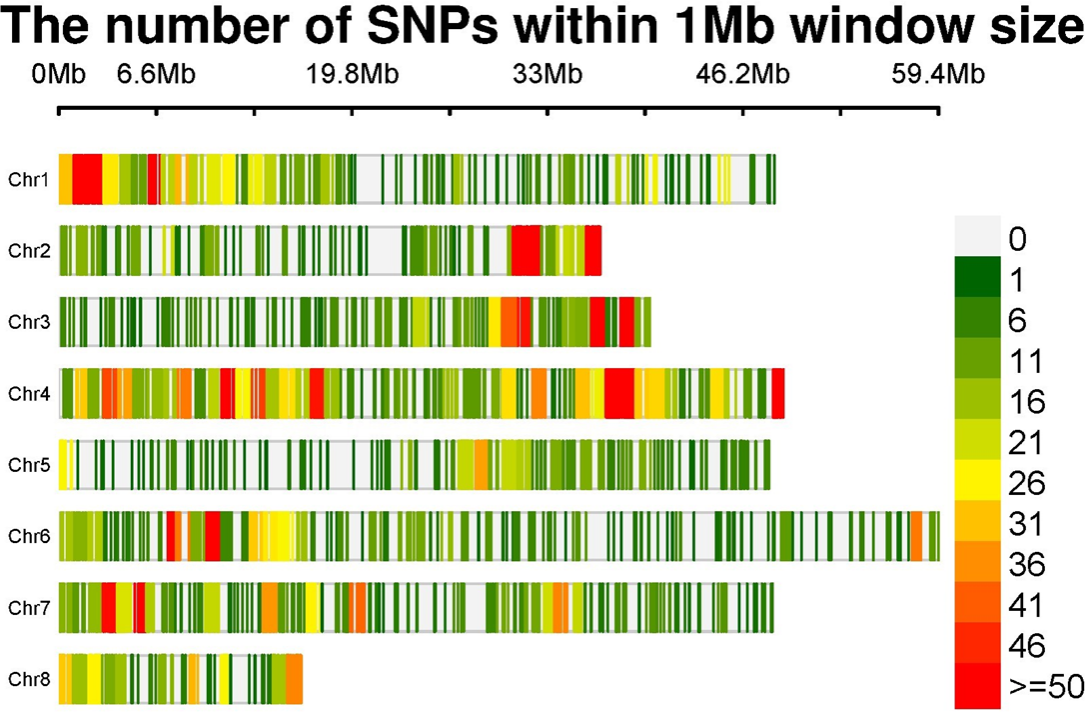
SNP density plot indicating the distribution of filtered SNPs across chromosomes. SNP, single-nucleotide polymorphism.

After initial analysis of 16,892 SNP markers across 153 genotypes, 4,530 SNP markers were retained after filtering. An in-depth analysis of genome-wide SNP markers revealed the following distribution across the genome: chromosome 1 (664 SNPs), chromosome 2 (469 SNPs), chromosome 3 (476 SNPs), chromosome 4 (1133 SNPs), chromosome 5 (342 SNPs), chromosome 6 (615 SNPs), chromosome 7 (579 SNPs), and chromosome 8 (252 SNPs) (**Table 3**). The numbers of SNP markers distributed over each chromosome are graphically depicted through an SNP markers density plot (**Figure 2**).

**Table 3:** Chromosome-wise SNP distribution in chickpea genome (n = 8).

Chromosome	Size (Mb)	16K SNP	4K SNP	NCBI CDC Frontier size (bp)	SNP distribution (per Mb)	LD decay (kb)
1	75.5	2,546	664	48,359,943	33.72185	111.9
2	71.4	1,516	469	36,634,854	21.23249	72.2
3	75.1	1,960	476	39,989,001	26.09854	168.8
4	86	3,204	1,133	49,191,682	37.25581	160.1
5	87.8	2,043	342	48,169,137	23.26879	13.6
6	99.5	2,617	615	59,463,898	26.30151	306.5
7	72.6	2,036	579	48,961,560	28.04408	118.8
8	77.4	970	252	16,477,302	12.5323	29.4
Whole genome	~740	16,892	4,530	530.8 (Mb)	22.82703	140.2

SNP, single-nucleotide polymorphism; LD, linkage disequilibrium.

### Analysis of SNP marker distribution, population diversity, and linkage disequilibrium

**Figure 3:**
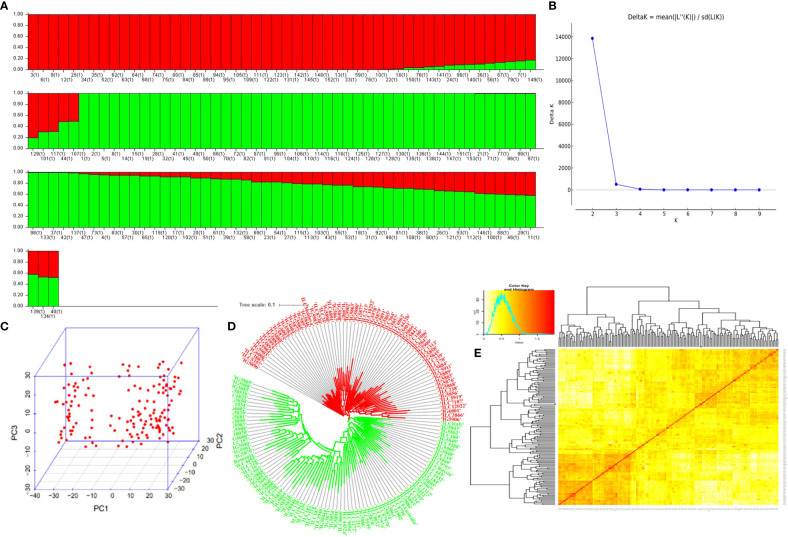
Population in the GWAS panel from model. **(A)** Population structure-based grouping of genotype from STRUCTURE analysis. **(B)** K *vs.* ΔK of structure harvest. **(C)** The 2D plot of the principal component-based grouping. **(D)** Neighbor-joining tree-based diversity. **(E)** Heat map of pairwise kinship matrix. GWAS, genome-wide association study.

**Figure 4:**
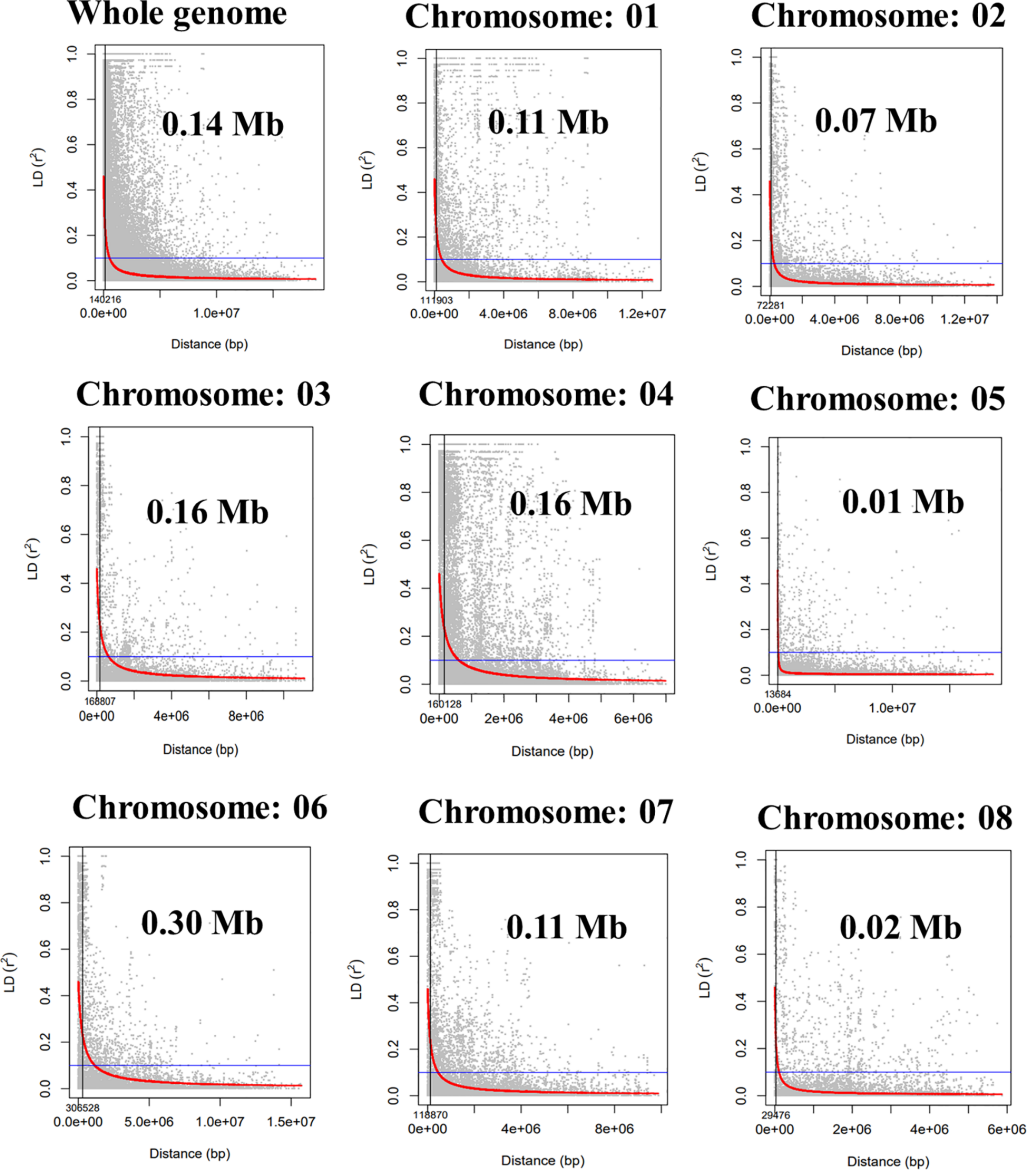
Whole-genome and individual chromosome-wide linkage disequilibrium (LD) decay in the GWAS panel. GWAS, genome-wide association study.

Population structure analysis revealed the best ΔK *vs.* K-value to be 2, indicating the presence of two distinct subpopulations within the GWAS panel (**Figures 3A, B**). Subpopulation 1 comprised 33.33% (51) of total genotypes, while Subpopulation 2 consisted of 50.32% (77) of total genotypes, and 16.33% (25) of total genotypes were classified as part of the admixture population (**Supplementary Table 3**). This finding was corroborated by PCA based on SNP marker data, which also depicted two distinct clusters (**Figure 3C**), reinforcing the presence of two subpopulations. Additionally, kinship and neighbor-joining cluster analyses also supported the presence of these two clusters (**Figures 3D, E**). LD between marker pairs was determined using r^2^ values, and LD decay plots were created by plotting r^2^ values against genetic distance in base pairs (bp). A significant LD block size of 0.14 Mb (140 kb) was observed across the entire genome, indicating that SNPs within this block exhibit strong linkage disequilibrium. For individual chromosomes, LD decay varied, with chromosome 5 displaying the highest decay at 0.01 Mb and chromosome 6 exhibiting a lower decay rate with a block size of 0.30 Mb (**Figure 4**).

### Genome-wide association study

**Figure 5:**
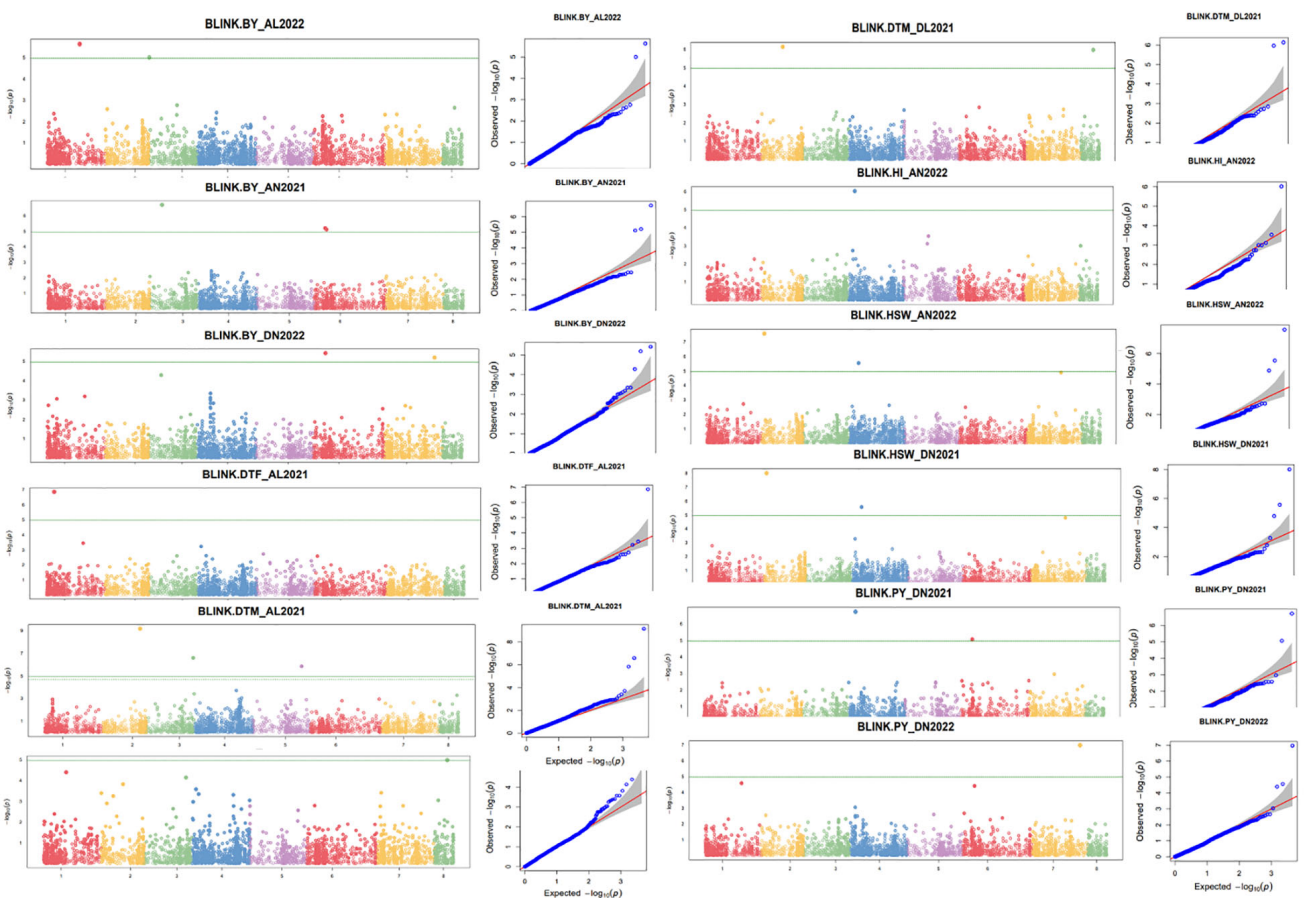
Manhattan and respective quantile–quantile (Q–Q) plots of significant associations for studied traits using individual BLUPs. BLUP, best linear unbiased prediction.

**Figure 6:**
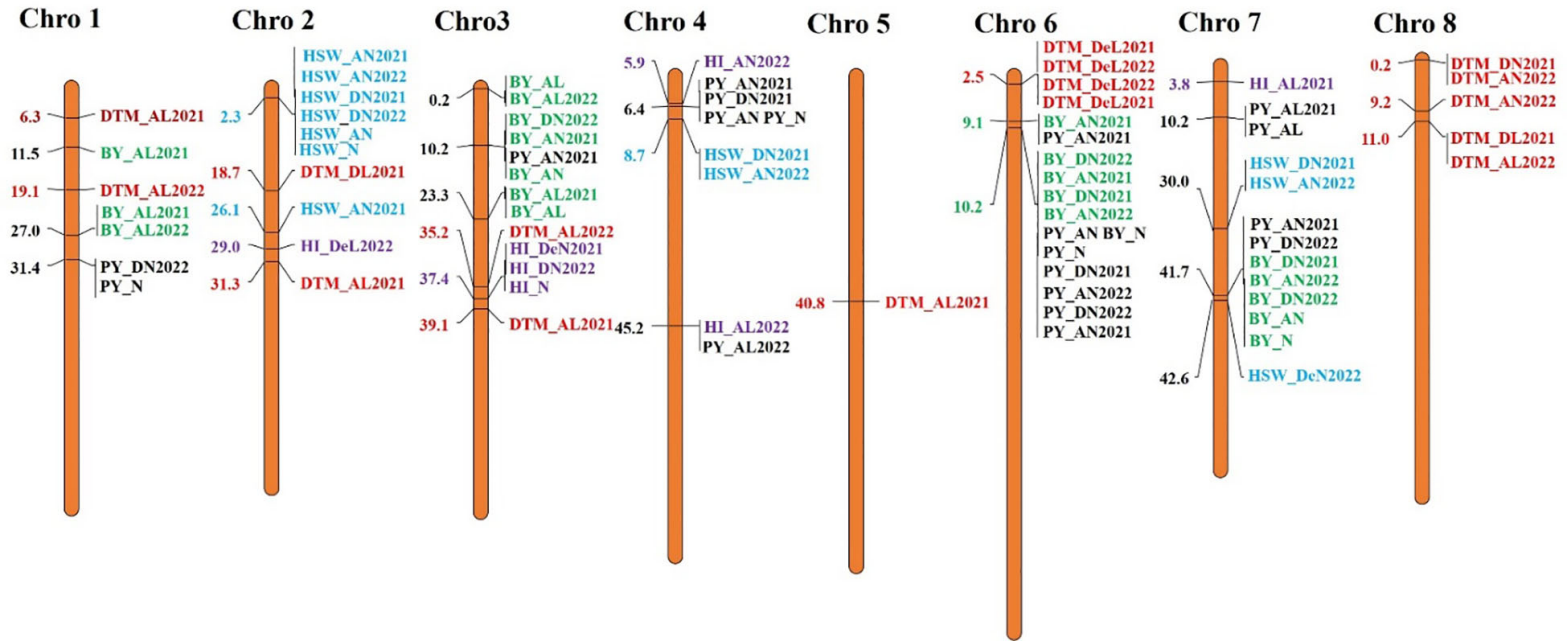
Distribution and position (in Mb) of identified marker–trait associations (MTAs) at their respective chromosome with associated traits over the seasons on chickpea chromosomes for plot yield (black), BY (green), DTF (brown), DTM (red), HI (purple), and HSW (cyan). BY, biomass yield; DTF, days to flowering; DTM, days to maturity; HI, harvest index; HSW, hundred-seed weight.

For the GWASs, BLUP values were analyzed for individual traits and conditions for both the years separately and combined across locations, years, BLUPs, and the overall BLUPs across treatments of locations. A total of 75 unique MTAs were identified above ≥−log_10_ (4.00) for all traits at a cut-off p-value of <0.05 as a Bonferroni correction for stringent selection [−log_10_(p) > 4.95]. After this correction, only 27 highly significant MTAs (**Table 4**; **Supplementary Table 4**) were found. Out of 27 MTAs, 10 MTAs for BY, one for DTF, six for DTM, two for HI, five for HSW, and three for PY were retained, and they were depicted using Manhattan and Q–Q plots (**Figure 5**; **Supplementary Figures 4A, B**). Common SNP was observed for marker Ca2:2311917 for HSW under Delhi 2021 under normal conditions, under Amlaha normal condition of season 2022, and Amlaha normal condition, which was combined BLUP of across location on chromosome 2 at 2.31-Mb location with −log_10_(p) ranging from 7.09 to 8. Additionally, another significant SNP, Ca4:8669498, was associated with HSW and was observed in both Amlaha 2022 and Dharwad 2021 under normal sown control conditions. The SNP Ca6:10230657 shows the pleiotropic effect for BY and PY on chromosome 6 at 10.23-Mb physical location with −log_10_(p) ranges from 5.06 to 6.44 under Dharwad 2022, Amlaha 2021, Dharwad 2022(PY) and by across-treatment BLUP (**Supplementary Figure 4B**). Similarly, pleiotropic effects and cross-confirmations were noted for SNPs Ca7:41673233 and Ca8:10963827 (**Supplementary Table 4**), while chromosomes 2, 3, and 6 had a maximum number of MTAs (6) to lowest (1) on chromosome 5 (**Figure 6**; **Supplementary Figure 5**).

**Table 4:** Significant marker–trait associations (MTAs) with a Bonferroni-corrected p-value [−log_10_(p) > 4.95] for traits under study at individual environment.

S.No.	Trait	SNP	Chromosome	Position	p-Value	−log_10_(p)
1	DTM_AL2021	Ca2:31252891	2	31252891	6.90E−10	9.161034
2	HSW_DN2021	Ca2:2311917	2	2311917	9.87E−09	8.005556
3	HSW_AN2022	Ca2:2311917	2	2311917	2.70E−08	7.568951
4	HSW_AN	Ca2:2311917	2	2311917	8.00E−08	7.097058
5	PY_DN2022	Ca7:41673233	7	41673233	1.09E−07	6.963979
6	DTF_AL2021	Ca1:6257653	1	6257653	1.41E−07	6.852291
7	PY_DN2021	Ca4:6352125	4	6352125	1.86E−07	6.730408
8	BY_AN2021	Ca3:10159944	3	10159944	1.97E−07	6.704917
9	DTM_AL2021	Ca3:39084979	3	39084979	2.58E−07	6.588161
10	BY_N	Ca6:10230657	6	10230657	3.59E−07	6.444971
11	DTM_DL2021	Ca2:18671666	2	18671666	7.33E−07	6.134748
12	HI_AN2022	Ca4:5907421	4	5907421	9.70E−07	6.013135
13	DTM_DL2021	Ca8:10963827	8	10963827	1.08E−06	5.967427
14	BY_N	Ca7:41673233	7	41673233	1.38E−06	5.860313
15	DTM_AL2021	Ca5:40828566	5	40828566	1.44E−06	5.840222
16	BY_AL2022	Ca1:27012660	1	27012660	2.34E−06	5.630731
17	HSW_DN2021	Ca4:8669498	4	8669498	2.73E−06	5.564497
18	HSW_AN2022	Ca4:8669498	4	8669498	2.90E−06	5.537057
19	BY_DN2022	Ca6:10230657	6	10230657	3.83E−06	5.417043
20	HI_N	Ca3:37444451	3	37444451	4.51E−06	5.34594
21	BY_AN2021	Ca6:9109096	6	9109096	6.25E−06	5.204069
22	BY_DN2022	Ca7:41673233	7	41673233	6.44E−06	5.190981
23	BY_AN2021	Ca6:10230657	6	10230657	7.72E−06	5.112628
24	PY_DN2021	Ca6:10230657	6	10230657	8.65E−06	5.062903
25	BY_AL	Ca3:23273262	3	23273262	9.40E−06	5.027069
26	BY_AL2022	Ca3:171579	3	171579	9.92E−06	5.003348
27	DTM_AL2022	Ca8:10963827	8	10963827	1.06E−05	4.97535

DTF, days to flowering (days); DTM, days to maturity (days); PH, plant height (cm); HSW, hundred-seed weight (g); BY, biological yield (g); HI, harvest index (%); PY, plot yield (g); SNP, single-nucleotide polymorphism.

### Allelic effects of identified genomic regions on respective phenotypes

**Figure 7:**
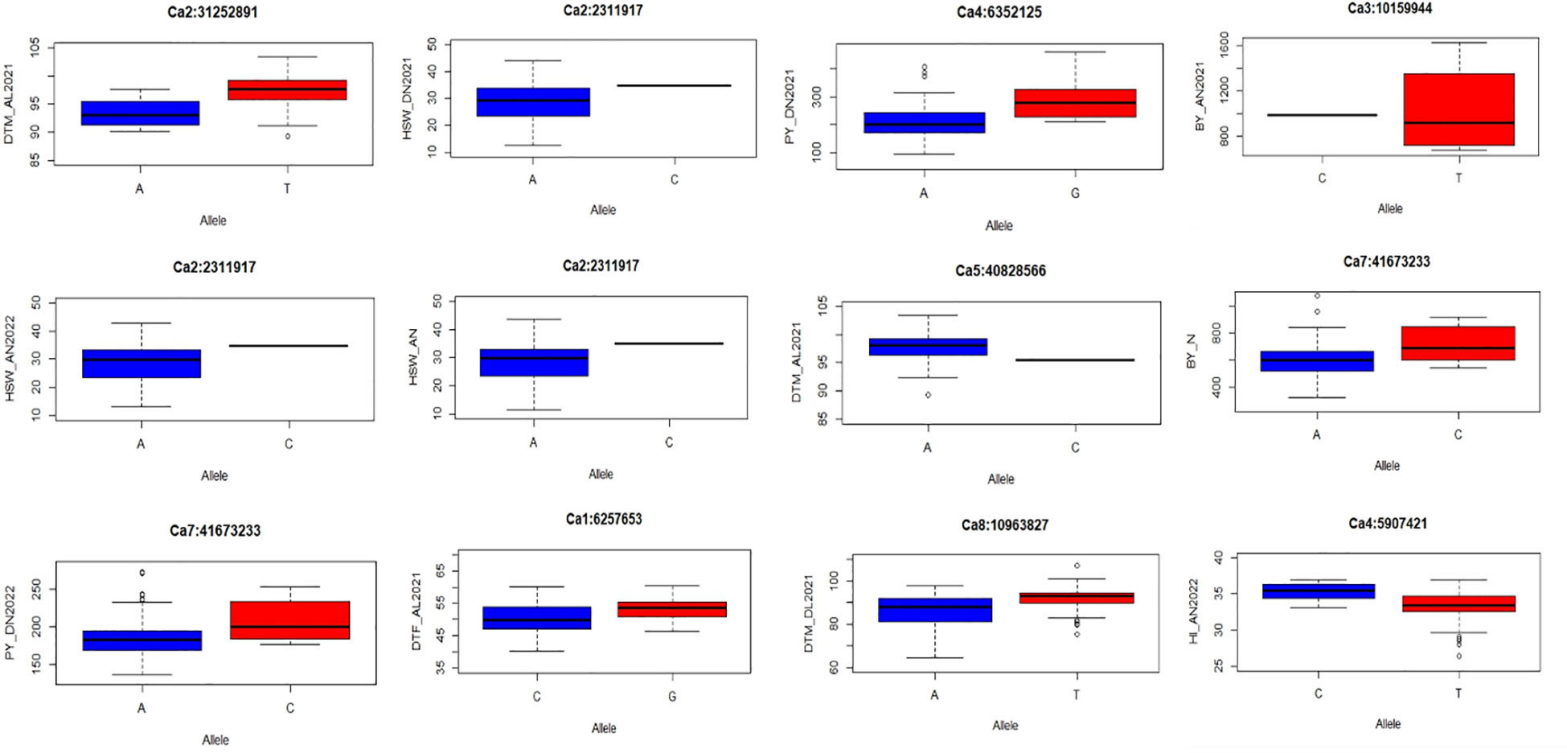
Allelic effects of selected MTAs identified in multiple locations for the studied traits under study. MTAs, marker–trait associations.

Twenty-seven major MTAs were analyzed to determine the range of phenotypic variations for all traits (**Figure 7**; **Supplementary Figure 6**). Boxplots display the phenotypic values associated with reliable QTNs that exhibit significant effects (p < 0.01) on their corresponding traits. The landraces should be categorized into two groups based on whether they carry the superior or inferior allele for each QTN. The x-axis of the plot represents the two allele types for each QTN, while the y-axis depicts the phenotypic values. Association panel genotypes were divided into two classes according to allele types. It was observed that all 27 QTNs demonstrated a significant effect on respective traits (p ≤ 0.01). Among these, marker Ca2:2311917 at Chr2 was pleiotropic for HSW_DN2021, HSW_AN2022, and HSW_AN. These significant associations suggest their plausible role in determining heat-tolerant traits.

### Putative candidate genes associated with MTAs

After rigorous selection criteria were applied, a total of 27 MTAs were identified and retained. To explore potential candidate genes associated with these MTAs, a search was conducted within a 100-kb region flanking each MTA considering LD decay of 140 kb. This search utilized sequence information obtained from the SNP markers and their genome position, which were identified through a BLAST search in PulseDB (Pulse Database) against the respective individual chromosomes of chickpeas. The identified marker Ca1:270126601 for trait BY_AL2022 at 27.01 Mb was found in or near probable coding regions that code for Protein MADS AFFECTING FLOWERING 5 of ortholog *Arabidopsis thaliana* and *Medicago truncatula* with Squamosa promoter-binding-like protein 12 and *S*-adenosylmethionine synthase ortholog of *C. arietinum*. Pentatricopeptide repeat-containing protein, subtilisin-like protease SDD1, and UDP-glycosyltransferase 43 also showed lineage with chickpeas (Zhang et al., 2020). Trait DTF_AL2021 of marker Ca1:6257653 codes for Cytochrome b561, DOMON domain-containing protein, auxin-induced in root cultures protein 12, heterogeneous nuclear ribonucleoprotein 1, and pentatricopeptide repeat-containing protein and also codes for Scarecrow-like protein 13. Another marker, Ca2:18671666, for trait DTM_DL2021 probably codes for GH3 auxin-responsive promoter and Fe(2+) transport protein 1. Marker Ca2:2311917 associated with HSW_DN2021, HSW_AN2022, and HSW_AN traits were annotated for linked genes deoxynucleoside triphosphate triphosphohydrolase SAMHD1 homolog and protein farnesyltransferase subunit beta ortholog of *Pisum sativum* and *Glycine max* (**Supplementary Data 1**).

## Discussion

Chickpea is an important dietary legume crop of arid and semi-arid regions and holds a prominent position as an economical source of protein-rich food. However, the potential yield of chickpeas faces significant challenges due to high-temperature stress at various growth stages. This stress has adverse effects on factors such as pollen viability, pollen germination on the stigma, pollen tube growth, and poor pod formation, ultimately affecting its yield potential. To combat these problems, it is imperative to gain a thorough understanding of the genomic regions that influence the heat-tolerant abilities of chickpeas. This knowledge is essential for developing specific varieties that are highly resilient to heat stress while exhibiting higher yields. In earlier studies, QTL-hotspot regions associated with drought tolerance and related traits were successfully introgressed to enhance the drought resistance of elite crop varieties through marker-assisted backcross (MABC) breeding (Bharadwaj et al., 2021). For such purposes, the identification of markers linked to the target traits is a fundamental requirement. Therefore, in this study, we sought to identify MTAs, linked to yield and yield-related traits under heat stress conditions, using germplasm collected from the WANA region.

Analysis of variance for all the studied traits (except HI under normal sown conditions in 2022) in each year indicated significant variability among these traits. This variability is a crucial prerequisite for conducting genetic studies and implementing breeding programs. Notably, there was significant genetic variability observed for the tested traits under both normal and heat stress conditions in both years (Jha et al., 2022). Higher trait values were observed in normal sown conditions as compared to late-sown conditions, such as DTF and DTM with the lowest mean values as stable across seasons and locations and higher values for all other traits studied considered under this study; for example, genotype IG5866 was stable under normal timely sown condition, or PY and ILC8666 were stable under late-sown condition (**Supplementary Table 6**). It is important to consider that PY and BY are complex traits characterized by a quantitative pattern of inheritance and susceptibility to environmental influences, including season and treatment due to soil nutrient conditions. Furthermore, when evaluating phenotypic characteristics, it was observed that the phenotypic coefficient of variation (PCV) and broad-sense heritability were notably higher for PY and BY when compared to the HI. Among the studied traits, DTM consistently exhibited high heritability across all 12 environments, followed by HSW (Hussain et al., 2021). It is worth noting that the heritability of PY varied from low to medium, spanning a wide range from 16.66% in Delhi under normal conditions in 2022 to 76% in Delhi under late conditions in 2021 (Paul et al., 2018). A significant positive correlation between grain yield and high heritability along with high genetic variability and fewer yield losses under optimal conditions are essential for a characteristic to be expressed as a heat-tolerant marker (Maqbool et al., 2017; Chandora et al., 2020). Interaction ANOVA showed a significant variation (p < 0.001) for all tested traits by considering genotype, treatment, season, replication, location, replication by block, genotype by location, genotype by treatment, treatment by location, and genotype by treatment by location. However, DTF, DTM, and HSW were non-significant for genotype by treatment by location and DTM for genotype by location, as these traits showed higher broad-sense heritability as near qualitative nature of traits, and traits DTM and HI were non-significant for replication within the block due to lower phenotypic variation for these traits influenced less by soil heterogeneity across location and treatments.

It was observed that Pearson’s correlation coefficient was found positive between PY, BY, and HI under all the treatments across the locations. Notably, PY showed a non-significant correlation with HSW and negative in Delhi 2022 late condition along with PH p < 0.05. This is because the association mapping (AM) panel predominantly consists of *kabuli* type, which is bold seeded, hence increasing the weight of seeds, and the costing number of seeds leads to independent contribution toward plot yield. Sandhu et al. (2012) observed a negative correlation that is due to the effect of heat stress hampering seed weight and ultimately yield. PCA shows that the component traits such as PY, BY, and HI predominantly contributed to PC1. These traits exhibited parallel trends and clustered together consistently across various locations and treatment conditions. Traits influenced by additive gene action and showing positive correlations can be collectively and efficiently improved irrespective of environmental influences (Borah et al., 2018).

GWAS panels were grouped into two sub-groups with 25 admixtures, clearly indicating the WANA region’s representativeness and relatedness. The LD decay over genetic distance in a population indicates the requirement of a marker density to capture the markers close enough to the causal loci (Flint-Garcia et al., 2003; Danakumara et al., 2021). A large LD block size of 0.14 Mb was found for the whole genome. However, a slow rate of LD decay was observed for chromosome 6 at 0.30 Mb followed by chromosomes 3 and 4 at 0.16 Mb size, whereas faster LD decay on chromosome 5 of 0.01 Mb size was observed (**Figure 4**) (Sokolkova et al., 2020). A higher LD was observed in chickpeas due to low effective recombination rates as compared to cross-pollinated crops (Niu et al., 2019; Samineni et al., 2022). The extent of LD decay in the association panel of chickpeas was observed at 200–300 kb (Basu et al., 2019) and 5 cM in the chickpea reference set (Thudi et al., 2014). The extent of LD can vary due to the complexity and size of the genome and marker number (Valdisser et al., 2017). The LD may vary in different populations because of population size, genetic drift, admixtures, selection, mutation, non-random mating, mode of pollination, gene-rich region, and recombination frequency (Vos et al., 2017).

A genome-wide association study was conducted using the BLINK model within the GAPIT package, which is considered superior for identifying QTNs and minimizing the occurrence of false positive associations as the most reliable method (Huang et al., 2019). A total of 27 significant MTAs were identified with a p-value threshold of <0.05. This rigorous approach was taken to increase the stringency of selection and reduce the likelihood of false positive results, and it involved applying a Bonferroni correction. In this analysis, a total of 22 individual markers were found to be significantly associated with the traits of interest (Kaler and Purcell, 2019). Recent advancements in the sequencing and annotation of the chickpea genome have provided valuable insights into the identification of candidate genes within the genomic regions pinpointed through GWAS. These candidate genes are believed to play a role in modulating the variation observed in heat-responsive traits (Srungarapu et al., 2022). We compiled a table of candidate genes located within 100-kb regions surrounding the identified MTAs, which included information such as their start and stop positions, InterPro IDs, gene ontology (GO) terms, and accessions, as well as the probable proteins they encode. In the genomic regions associated with linked SNP markers for PY, an MTA on chromosome 3 at 15.5 Mb was previously reported by Kalve et al. (2022). However, the remaining MTAs were novel findings specific to our study. For instance, SNP marker Ca1:27012660, linked to BY_AL2022, was located near candidate regions encoding a sucrose non-fermenting 4-like protein ortholog of *A. thaliana*. Through *in silico* analysis, we identified coding regions in the vicinity of Ca1:27012752 to Ca1:27025275 on chromosome 1. These regions also encoded a pentatricopeptide repeat-containing protein orthologous to *A. thaliana*. This protein has been recognized as important for resistance against both biotic and abiotic stresses in *Oryza sativa* (Qiu et al., 2021). Moreover, mQTL-seq analysis revealed a functionally relevant candidate gene within a major QTL region governing pod number in chickpeas. This gene belongs to the pentatricopeptide repeat (PPR) family and exhibits over 80% sequence conservation with its Arabidopsis ortholog, At1g52620 (Das et al., 2016). Additionally, our investigation identified genes such as pentatricopeptide repeat-containing proteins and serine threonine protein kinases, both of which have well-established roles in seed development across various crop species (Li et al., 2014). These findings shed light on the potential involvement of these genes in stress management and nutrient content regulation within chickpea grains.

The MTA associated with DTM for the year 2021 is linked to marker Ca2:18671666. This marker likely encodes the indole-3-acetic acid–amido synthetase GH3.1 ortholog, which has similarities to its counterparts in *A. thaliana* and *Solanum lycopersicum*. Furthermore, expression profiling studies have indicated the diverse roles of GH3 genes in various aspects of development and responses to abiotic stress in leguminous crops like chickpeas. Notably, genes such as CaGH3-3 in chickpeas; GmGH3-8 and GmGH3-25 in soybean; and LjGH3-4, LjGH3-5, LjGH3-9, and LjGH3-18 in *Lotus* were found to be upregulated in root tissues. This suggests their potential involvement in root development processes. Moreover, certain GH3 genes, specifically CaGH3-1 and CaGH3-7 in chickpeas, as well as MtGH3-7, MtGH3-8, and MtGH3-9 in *Medicago*, exhibited high levels of induction under conditions of drought and/or salt stress (Singh and Jain, 2014). A common MTA for HSW_HSW_DN2021, HSW_AN2022, and HSW_ANP is a putative disease resistance protein ortholog of *Solanum bulbocastanum* and *M. truncatula* with the marker location ranging from Ca2.2322855 to Ca2.2323917, which is also encoded to deoxynucleoside triphosphate triphosphohydrolase, SAMHD1 homolog, ortholog of *Dictyostelium discoideum*, and *G. max* effects on the control of cell proliferation and apoptosis (Felip et al., 2022). Trait DTM_AL2021 with marker Ca2:31252891 association encodes cation/H(^+^) antiporter 15 ortholog of *A. thaliana* and *M. truncatula* role as universal stress protein domain containing drought-responsive genes in pigeon pea (*Cajanus cajan* L.) (Sinha et al., 2015). Trait BY_AN2021 with marker Ca3:10159944 encodes probably inactive leucine-rich repeat receptor-like protein kinase role (LRR-RLK) as represents the largest group of RLKs in plants and plays vital roles in plant growth, development, and the responses to environmental stress (Song et al., 2022). Trait BY_AL2022 of SNP Ca3:171579 encodes to tetraketide alpha-pyrone reductase 1 ortholog of *A. thaliana* and dihydroflavonol-4-reductase as ortholog of *M. truncatula* and has a role in fatty acyl-CoA ester synthesis. Trait BY_AL is associated with marker Ca3:23273262 encoded by ATP-dependent helicase and HI_N marker Ca3:37444451 that encodes for aspartic proteinase ortholog sequence of *O. sativa* subsp. *japonica* and *M. truncatula* as reported recently by Varshney et al. (2019) using a dataset of 3.65 million SNPs derived from the resequencing of 429 chickpea germplasms collected globally. Additionally, several potential candidate genes were highlighted in their investigation, including TIC, REF6, aspartic protease, cc-NBS-LRR, and RGA3. These genes were found to play crucial roles in conferring tolerance to both heat and drought stress conditions. Furthermore, the identified MTAs and genomic regions were recognized as stable controllers of traits related to pods per plant, yield, and phenological traits (Rani et al., 2020). Trait DTM_AL2021 with SNP Ca3:39084979 marker linked to AT-hook motif nuclear-localized protein 14 role as AT-hook motif nuclear localized (AHL) gene family is a highly conserved transcription factor critical for the growth, development, and stress tolerance of plants (Zhang et al., 2023). Marker Ca4:5907421 for trait HI_AN2022 codes likely proteins as dormancy-associated protein homolog 3 ortholog of *A. thaliana* and *M. truncatula*, as its over-expression of glucose-6-phosphate/phosphate translocator 2 (GPT2) was investigated in chickpea leaves with roles as heat tolerance. Also, transgenic *A. thaliana* over-expressing metallothionein 1 (MT1) gene of desi chickpea was subjected to transcriptome analysis, and it drought tolerance was evaluated in 7-day-old plants (Kumar et al., 2022). Trait PY_DN2021 with SNP Ca4:6352125 relates to glycogen synthase kinase-3 homolog and shows genome-wide identification and expression analysis of glycogen synthase kinase encoding genes in foxtail millet (*Setaria italica* L.) under salinity, dehydration, and oxidative stress (Singh et al., 2023). Marker Ca4:8669498 for HSW_DN2021 and HSW_AN2022 codes for probable disease resistance RPP8-like protein 4. Similarly, trait DTM_AL2021 with marker Ca5:40828566 codes for methionine gamma-lyase ortholog sequence of *A. thaliana* and cystathionine gamma-lyase of ortholog *M. truncatula*; methionine gamma lyase maintains the equilibrium of isoleucine in a variety of plants under different environmental conditions such as drought (Joshi and Jander, 2009; Khan et al., 2019). Pleiotropic marker Ca6:10230657 for traits BY_N, BY_DN2022, BY_AN2021, and PY_DN2021, which codes serine/threonine-protein phosphatase PP2A catalytic subunit, is involved in several physiological responses in plants, playing important roles in developmental programs, stress responses, and hormone signaling. Six PP2A catalytic subunits (StPP2Ac) were identified in cultivated potato (Muñiz García et al., 2022). Trait BY_AN2021 associated with marker Ca6:9109096 codes for magnesium transporter MRS2-4A root-expressed magnesium transporter of the MRS2/MGT gene family in *A. thaliana* and allows for growth in low-Mg^2+^ environments (Gebert et al., 2009). Also, trait BY_AN2021 with marker Ca6:9109096 codes for 2Fe-2S ferredoxin-type domain participates in numerous biological processes, including carbon fixation, nitrogen assimilation, chlorophyll metabolism, and fatty acid synthesis, and it contributes to plant resilience against heat stress (Meng et al., 2022). Traits PY_DN2022, BY_N, and BY_DN2022 with marker Ca7:41673233 codes for β-galactosidase-like protein ortholog of *M. truncatula*-related proteins, including β-galactosidase, glucanase, sucrose synthase, cystathionine gamma-synthase, 1-aminocyclopropane-1-carboxylic acid oxidase, abscisic acid β-glucosyltransferase, and late embryogenesis abundant proteins, which all impart heat stress tolerance in chickpeas (Parankusam et al., 2017). Ca7:41673233 also codes for phosphatidylinositol-4-phosphate 5-kinase, and its role as heat shock triggers phospholipid-based signaling pathways (Yadav et al., 2017).Traits DTM_DL2021 and DTM_AL2022 of marker Ca8:10963827 code for pentatricopeptide repeat-containing protein ortholog sequence of *A. thaliana* and *M. truncatula* function as exposure to heat stress; the gene expression pattern was significantly altered in relation to various key factors such as heat shock proteins (HSPs), ubiquitin-protein ligases, transcription factors, and pentatricopeptide repeat-containing proteins. Notably, several genes were found to exhibit significant changes in their expression levels. Specifically, genes encoding heat shock proteins (CL2311.Contig3 and CL6612.Contig2), cytochrome P450 enzymes (CL4517.Contig4 and CL683.Contig7), and basic helix-loop-helix transcription factors (bHLH TFs) (CL914.Contig2 and CL8321.Contig1) showed distinctive induction patterns following 4 days of exposure to heat stress (Wang et al., 2019). The candidate regions we investigated contained a significant number of genes, most of which have been recognized for their vital contributions to plant development. Many of these genes have closely related counterparts in other species, and extensive research on these counterparts has provided valuable insights into their functions. Our analysis, which included assessing gene expression and applying gene ontology, pinpointed specific genes with elevated expression levels, shedding light on their roles within cellular organelles. To gain a deeper knowledge of this genomic region and its potential significance, future research could delve into detailed investigations of each identified region.

## Conclusion

Past efforts have successfully utilized drought-related QTL-hotspot regions to enhance drought tolerance in elite chickpea varieties through marker-assisted backcross breeding. To achieve this, the identification of markers linked to the trait of interest is crucial. In this study, we employed a diverse mapping panel to identify MTAs related to yield and yield-related traits under heat stress. Our analyses revealed significant genetic variability for the tested traits under both normal and heat stress conditions, laying the foundation for genetic studies and breeding programs. Phenotypic coefficients of variation and broad-sense heritability were relatively high for yield-related traits, indicating their suitability as potential heat tolerance markers. Additionally, we observed significant interactions between genotypes, treatments, seasons, locations, and other factors, reflecting the complex nature of these traits. Pearson’s correlation coefficients indicated positive relationships between yield traits and harvest index across most treatments and locations. However, we observed some negative correlations due to specific seed characteristics and the impact of heat stress on seed development. Principal component analysis highlighted the importance of yield-related traits in explaining variation across locations and treatments. The LD analysis demonstrated a slow LD decay across the chickpea genome, indicating extended LD blocks and low effective recombination rates, which is a characteristic of self-pollinated crops. Through GWASs using the BLINK model, we identified 27 significant MTAs linked to various traits associated with heat stress tolerance. These MTAs can serve as valuable targets for further research and breeding efforts. Notably, we identified candidate genes near these MTAs, and we further explored candidate genes situated near these MTAs, shedding light on the molecular mechanisms governing heat stress tolerance and yield enhancement in chickpeas such as indole-3-acetic acid–amido synthetase GH3.1 with GH3 auxin-responsive promoter and pentatricopeptide repeat-containing protein. These identified MTAs and associated candidate genes serve as valuable assets for breeding programs dedicated to crafting resilient chickpea varieties in the face of climate change. Trait HI associated with marker Ca3:37444451 encodes aspartic proteinase ortholog sequence of *O. sativa* subsp. *japonica* and *M. truncatula*, contributing to heat and drought tolerance as stable significant MTAs/genomic regions controlling yield trait. These genes have the potential to be integrated into the well-performing but heat- and drought-sensitive popular chickpea cultivars, which ultimately contribute to improved chickpea crop performance under adverse environmental conditions. The insights gained pave the way for future breeding endeavors, ultimately contributing to global food security.

## Data availability statement

The original contributions presented in the study are included in the article/**Supplementary Material**. Further inquiries can be directed to the corresponding authors.

## Author contributions

TD: Data curation, Formal analysis, Investigation, Software, Writing – original draft. NK: Data curation, Formal analysis, Investigation, Writing – review & editing. BP: Data curation, Formal analysis, Investigation, Supervision, Writing – review & editing. TK: Data curation, Investigation, Methodology, Writing – review & editing. CB: Conceptualization, Funding acquisition, Investigation, Methodology, Project administration, Software, Supervision, Visualization, Writing – original draft, Writing – review & editing. PJ: Data curation, Software, Writing – review & editing. MSN: Data curation, Software, Validation, Writing – review & editing. NJ: Formal analysis, Investigation, Methodology, Writing – review & editing. SP: Data curation, Software, Validation, Writing – review & editing. SB: Investigation, Methodology, Writing – review & editing. CK: Visualization, Writing – review & editing. RV: Formal analysis, Methodology, Software, Visualization, Writing – review & editing.
