# Supplementary material for: Unraveling the genetics of heat tolerance in chickpea landraces (Cicer arietinum L.) using genome-wide association studies
Source: Front Plant Sci. 2024 Mar 25;15:1376381. doi: 10.3389/fpls.2024.1376381 (PMC10999645; doi:10.3389/fpls.2024.1376381)

Supplementary Figures

Figure S1: Trait wise Frequency distribution of all the studied traits of each environment

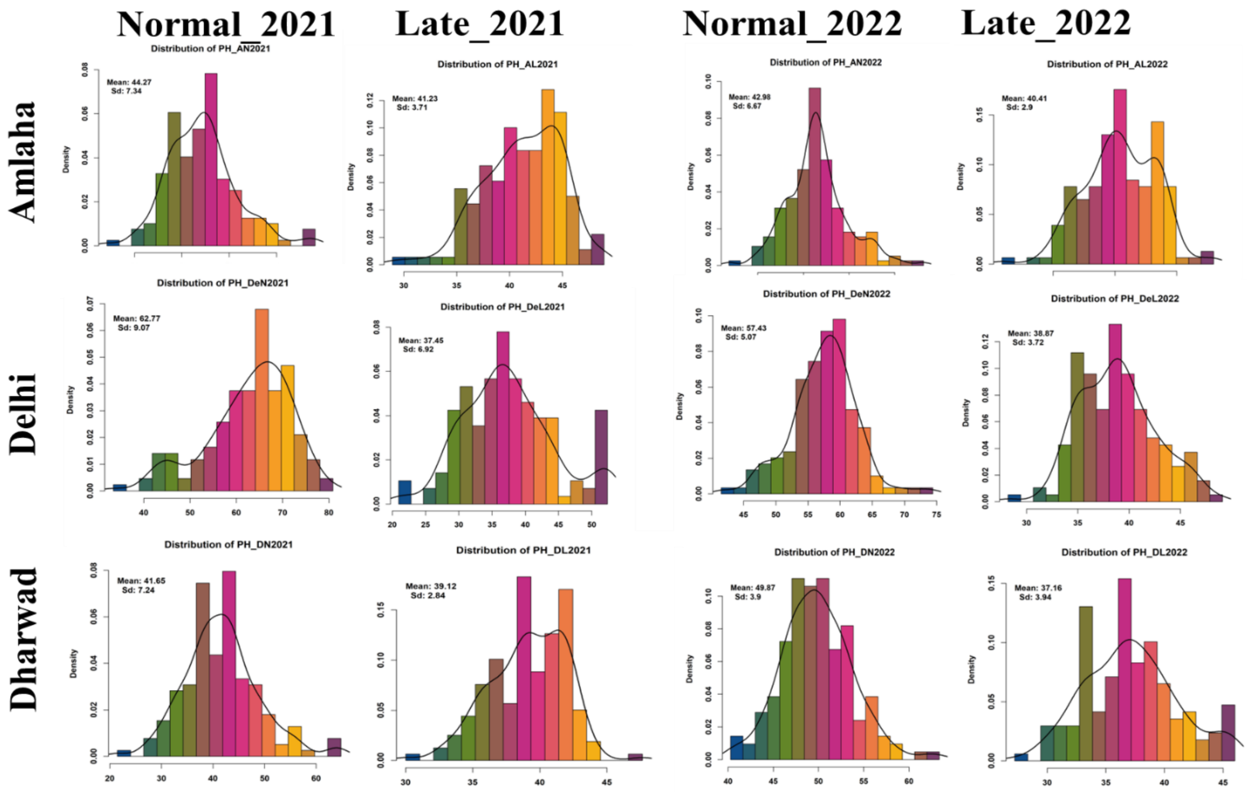

1. Plant Height (PH)

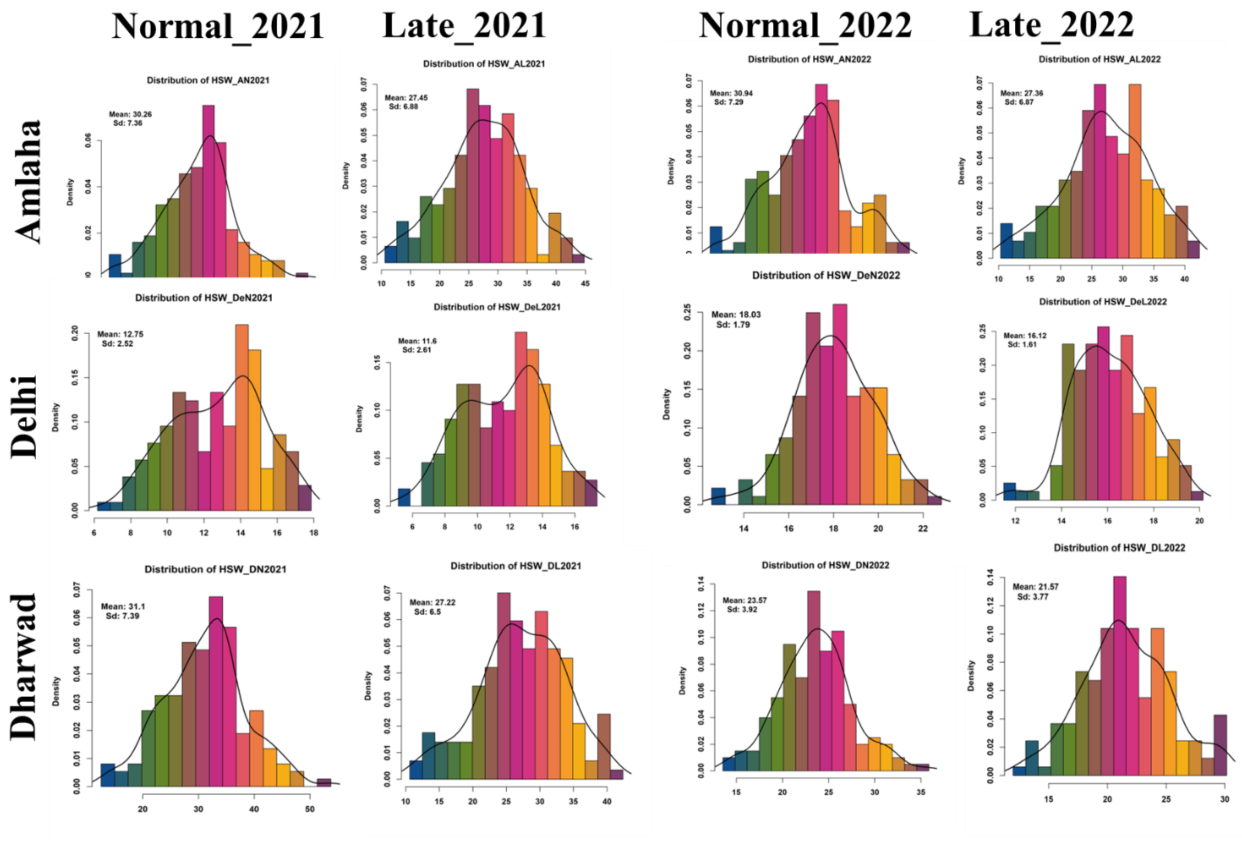

2. 100SW (HSW)

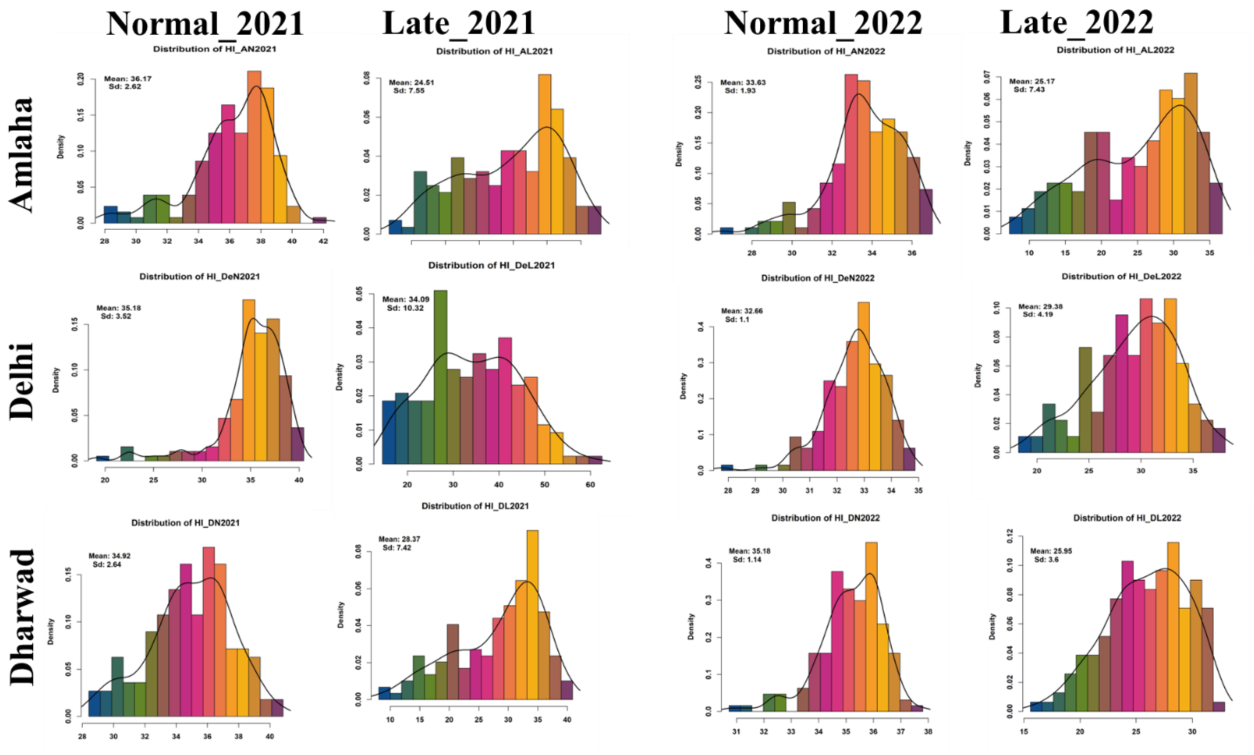

3. Harvest Index (HI)

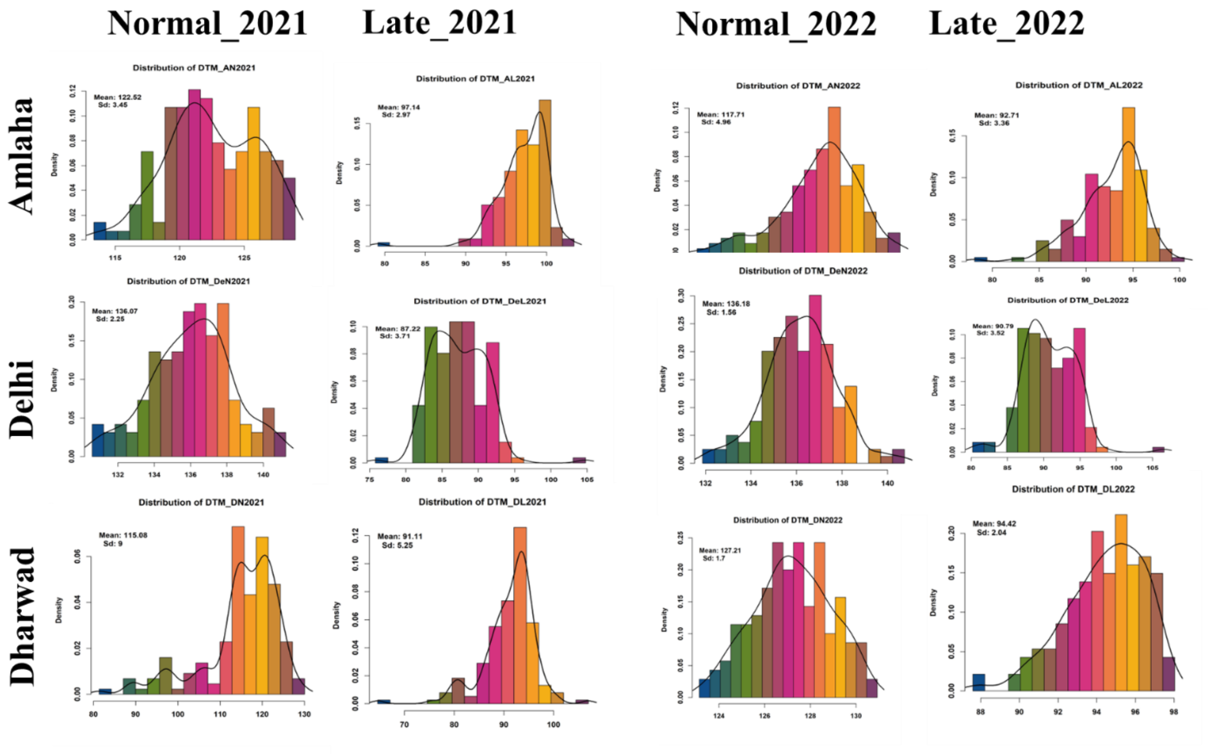

4. Days To Maturity (DTM)

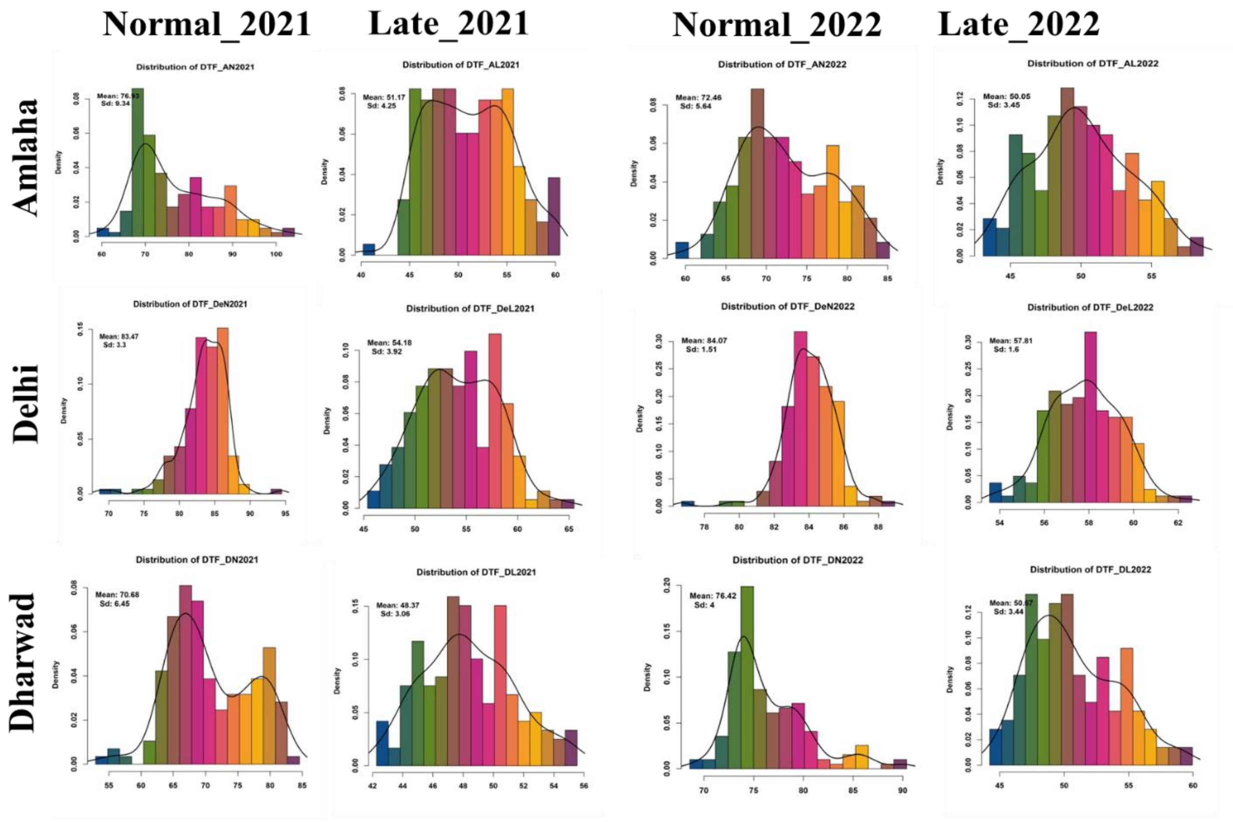

5. Days To Flowering (DTF)

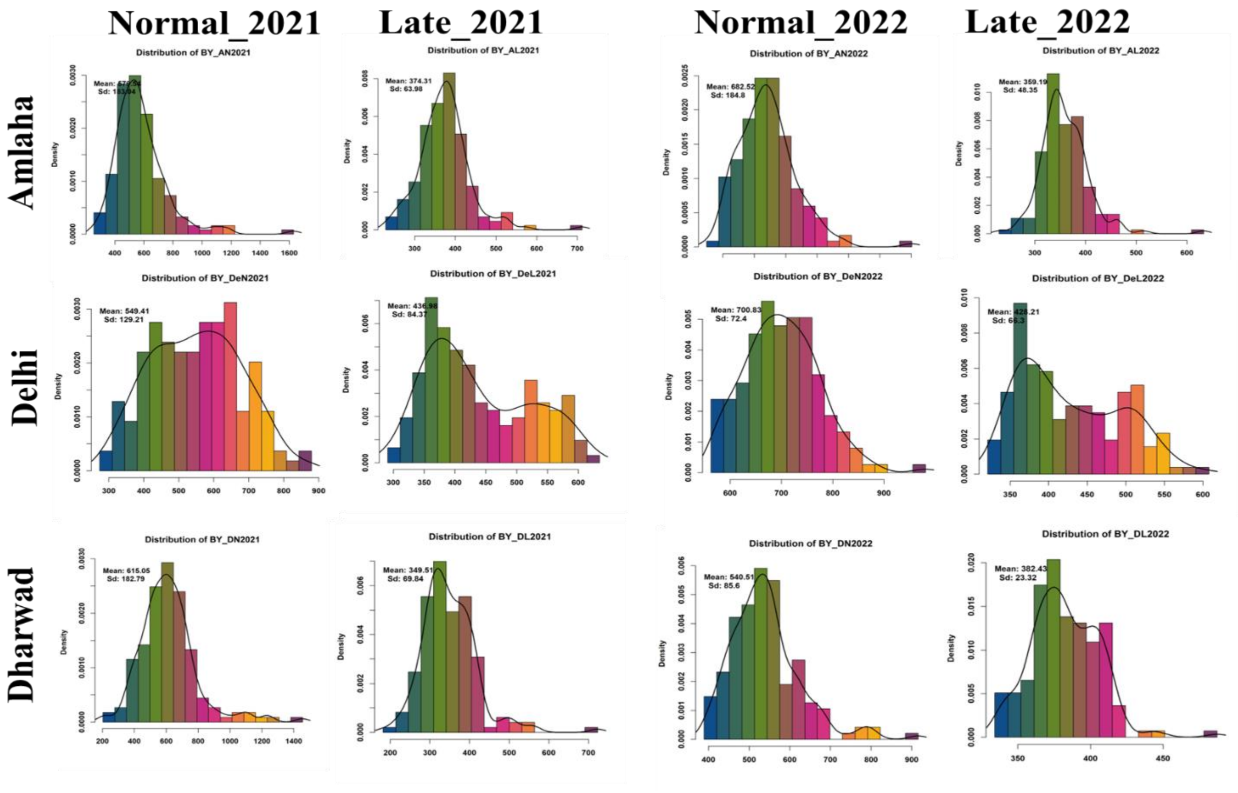

6. Biological Yield (BY)

Figure S2. Plots showing the pictorial representation of correlation among the studied traits at each environment.

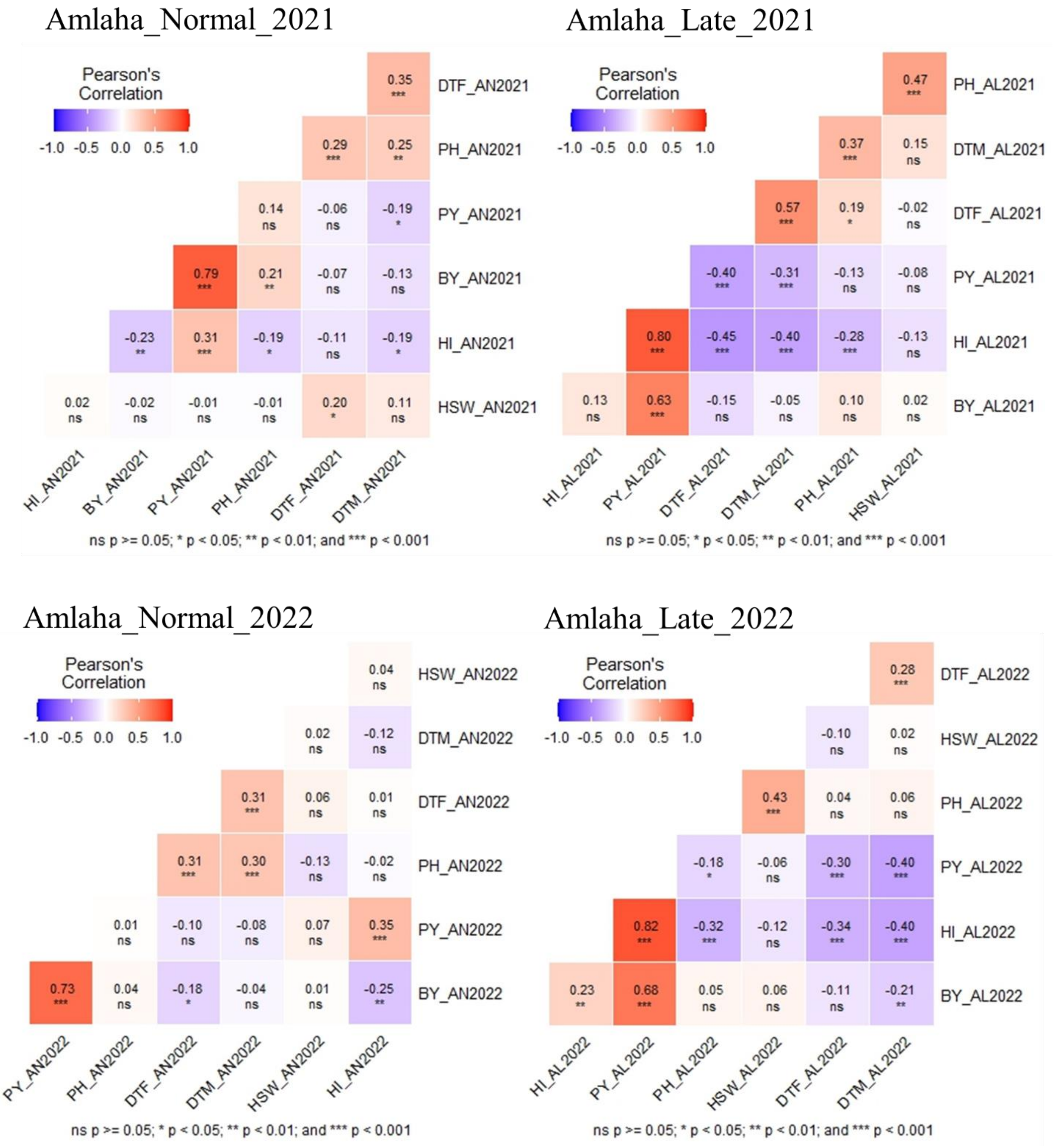

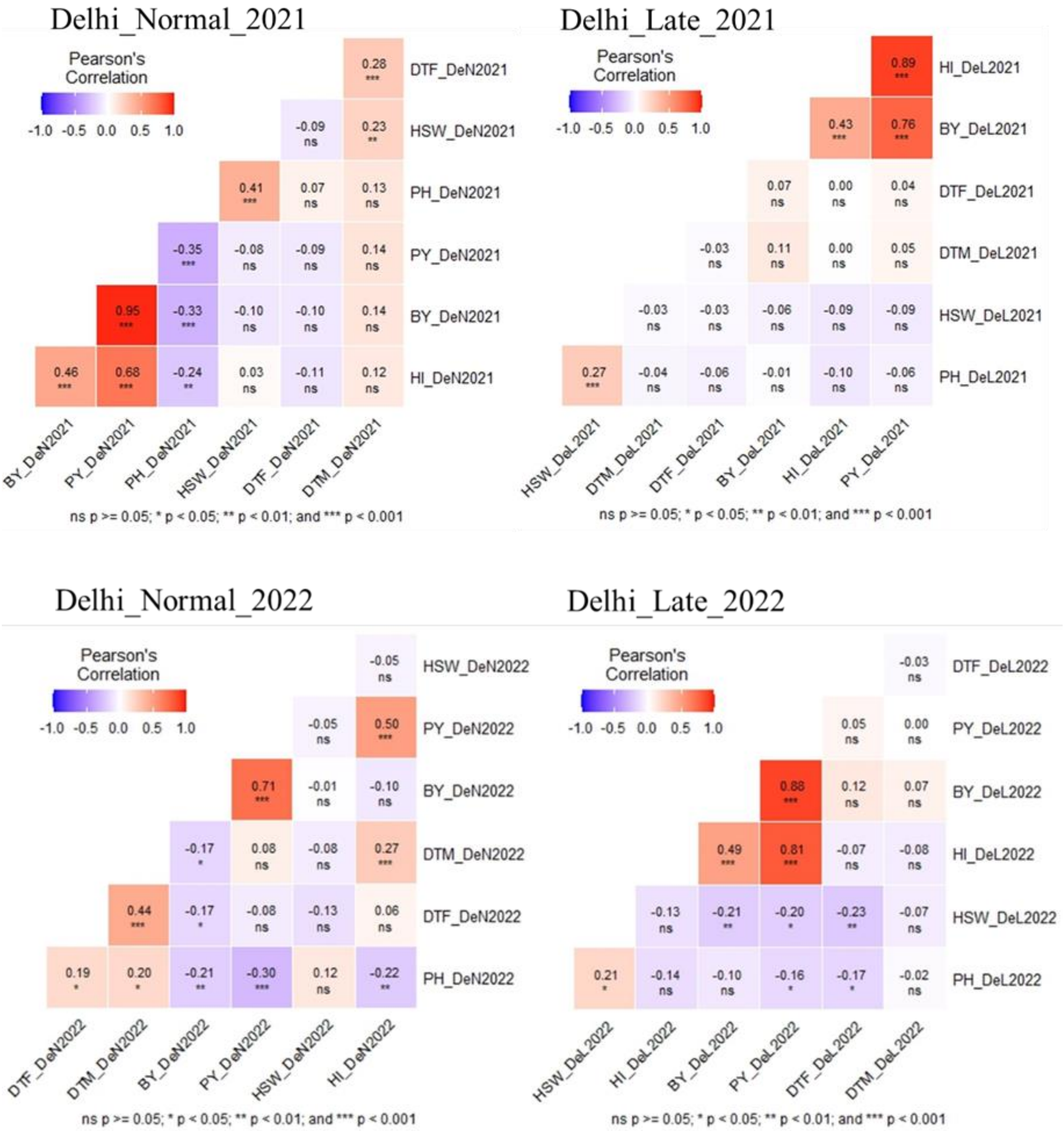
Delhi_Normal_2021
Delhi_Late_2021
Delhi_Normal_2022
Delhi_Late_2022
Pearson's Correlation
ns p >= 0.05; * p < 0.05; ** p < 0.01; and *** p < 0.001

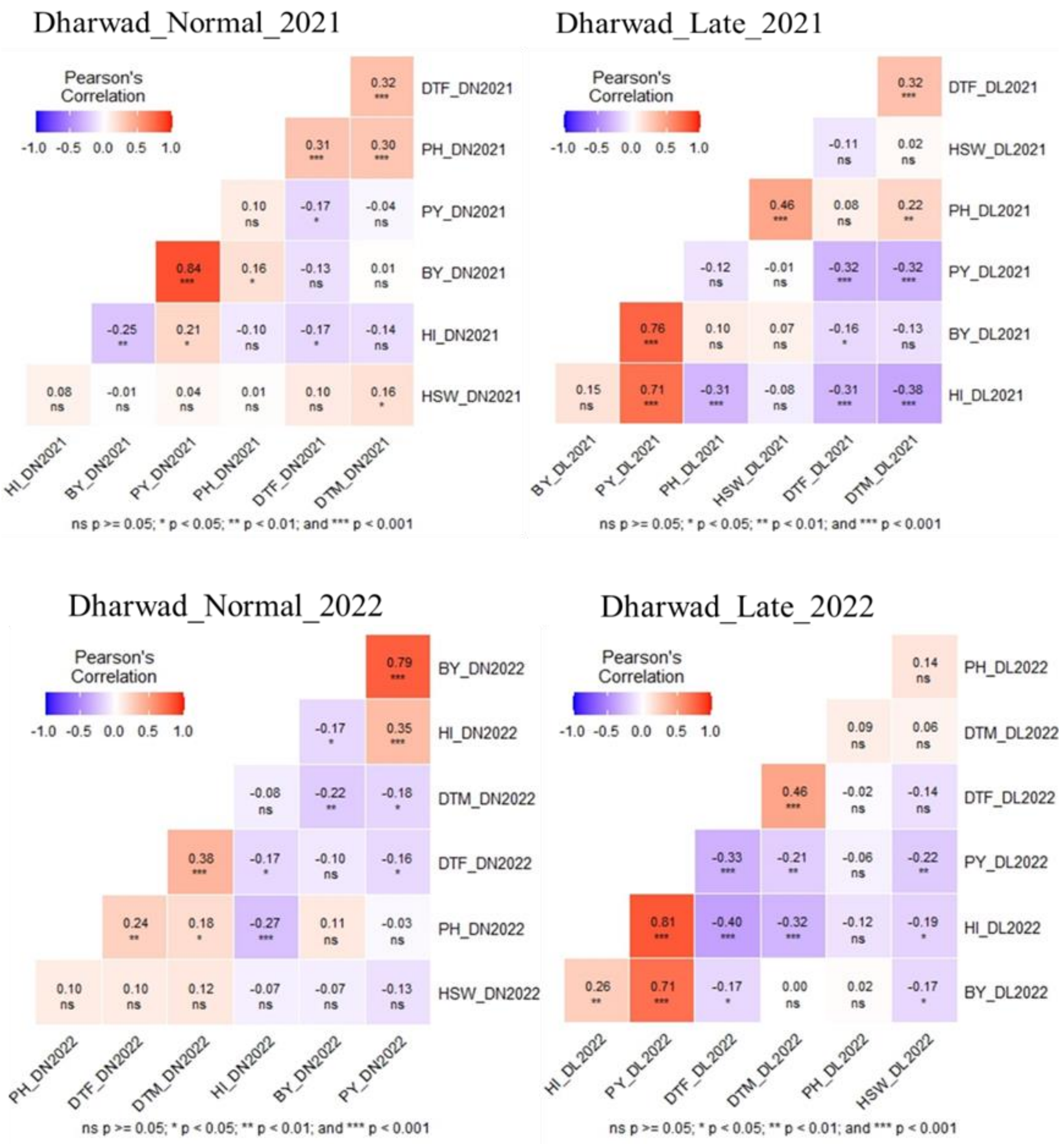
Dharwad_Normal_2021
Pearson's Correlation
-1.0 -0.5 0.0 0.5 1.0
DTF_DN2021: 0.32 ***
PH_DN2021: 0.31 *** 0.30 ***
PY_DN2021: 0.10 ns -0.17 * -0.04 ns
BY_DN2021: 0.84 *** 0.16 * -0.13 ns 0.01 ns
HI_DN2021: -0.25 ** 0.21 * -0.10 ns -0.17 * -0.14 ns
HSW_DN2021: 0.08 ns -0.01 ns 0.04 ns 0.01 ns 0.10 ns 0.16 *
HI_DN2021 BY_DN2021 PY_DN2021 PH_DN2021 DTF_DN2021 DTM_DN2021
ns p >= 0.05; * p < 0.05; ** p < 0.01; and *** p < 0.001
Dharwad_Late_2021
Pearson's Correlation
-1.0 -0.5 0.0 0.5 1.0
DTF_DL2021: 0.32 ***
HSW_DL2021: -0.11 ns 0.02 ns
PH_DL2021: 0.46 *** 0.08 ns 0.22 **
PY_DL2021: -0.12 ns -0.01 ns -0.32 *** -0.32 ***
BY_DL2021: 0.76 *** 0.10 ns 0.07 ns -0.16 * -0.13 ns
HI_DL2021: 0.15 ns 0.71 *** -0.31 *** -0.08 ns -0.31 *** -0.38 ***
BY_DL2021 PY_DL2021 PH_DL2021 HSW_DL2021 DTF_DL2021 DTM_DL2021
ns p >= 0.05; * p < 0.05; ** p < 0.01; and *** p < 0.001
Dharwad_Normal_2022
Pearson's Correlation
-1.0 -0.5 0.0 0.5 1.0
BY_DN2022: 0.79 ***
HI_DN2022: -0.17 * 0.35 ***
DTM_DN2022: -0.08 ns -0.22 ** -0.18 *
DTF_DN2022: 0.38 *** -0.17 * -0.10 ns -0.16 *
PH_DN2022: 0.24 ** 0.18 * -0.27 *** 0.11 ns -0.03 ns
HSW_DN2022: 0.10 ns 0.10 ns 0.12 ns -0.07 ns -0.07 ns -0.13 ns
PH_DN2022 DTF_DN2022 DTM_DN2022 HI_DN2022 BY_DN2022 PY_DN2022
ns p >= 0.05; * p < 0.05; ** p < 0.01; and *** p < 0.001
Dharwad_Late_2022
Pearson's Correlation
-1.0 -0.5 0.0 0.5 1.0
PH_DL2022: 0.14 ns
DTM_DL2022: 0.09 ns 0.06 ns
DTF_DL2022: 0.46 *** -0.02 ns -0.14 ns
PY_DL2022: -0.33 *** -0.21 ** -0.06 ns -0.22 **
HI_DL2022: 0.81 *** -0.40 *** -0.32 *** -0.12 ns -0.19 *
BY_DL2022: 0.26 ** 0.71 *** -0.17 * 0.00 ns 0.02 ns -0.17 *
HI_DL2022 PY_DL2022 DTF_DL2022 DTM_DL2022 PH_DL2022 HSW_DL2022
ns p >= 0.05; * p < 0.05; ** p < 0.01; and *** p < 0.001

Figure S3. Environment wise PCA Biplots from the traits under investigation

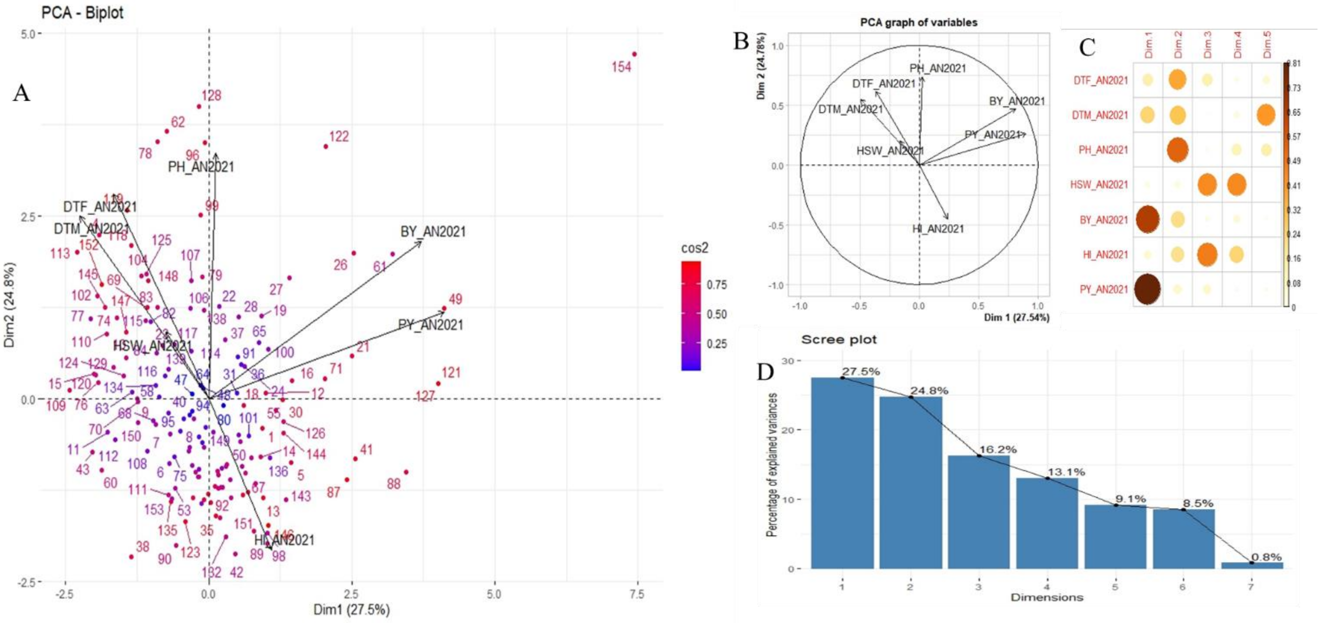

Supplementary Figure 3. Amla timely 2021

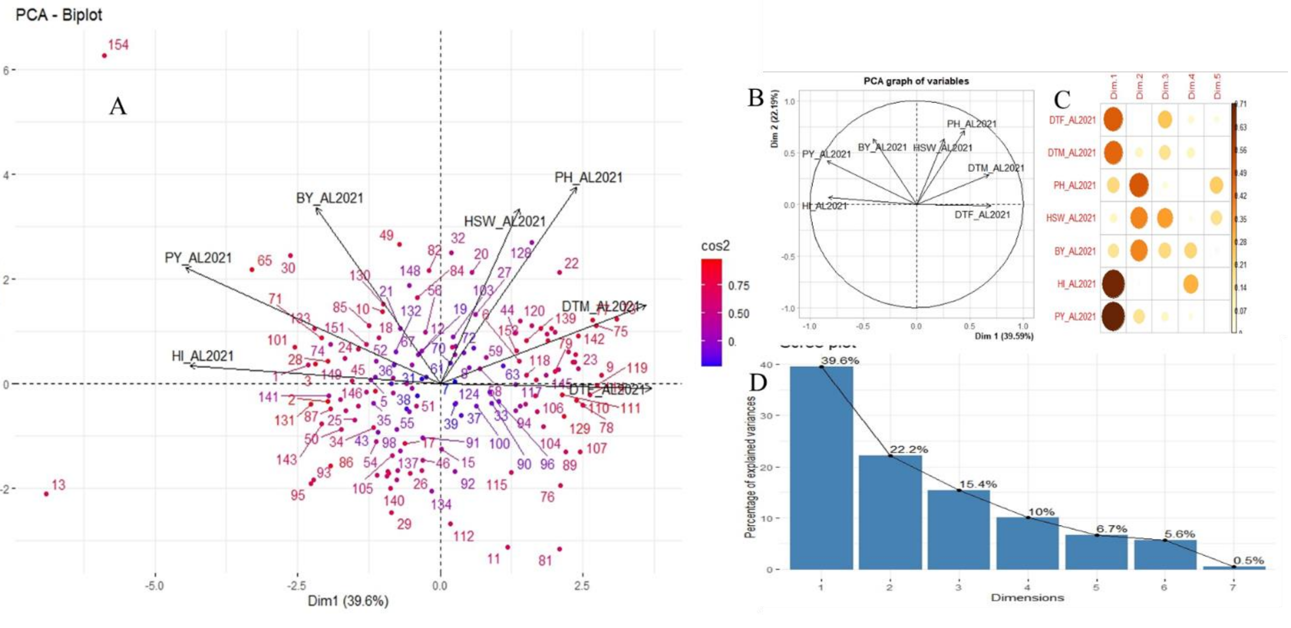

Supplementary Figure 3. Amla late 2021

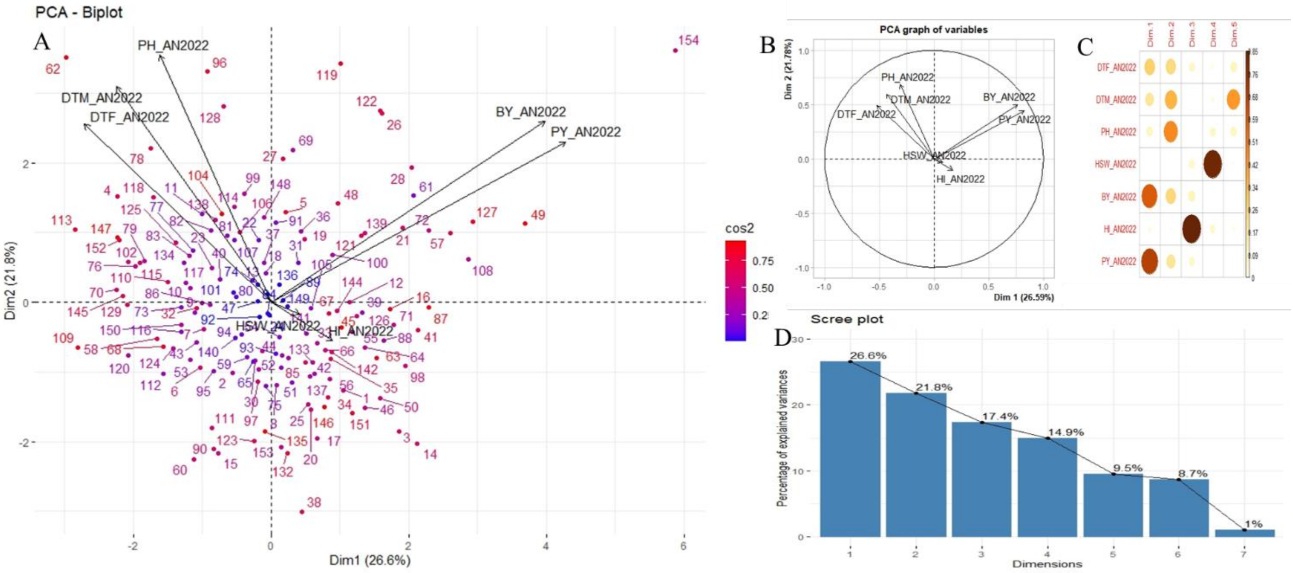

Supplementary Figure 3. Amla timely 2022

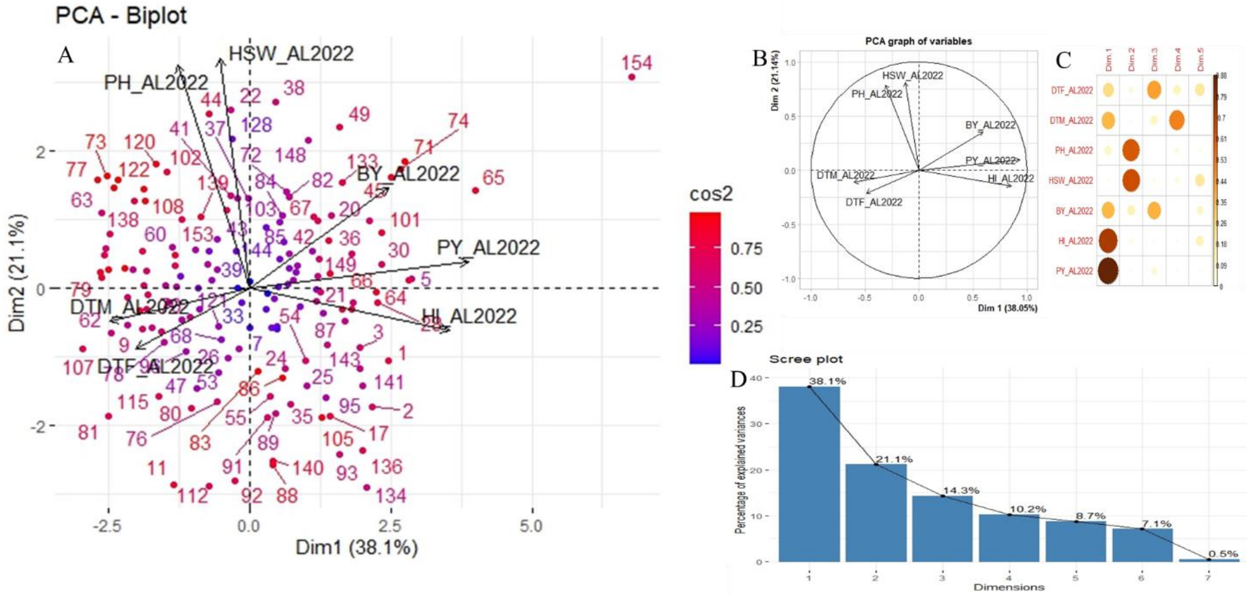

Supplementary Figure 3. Amla late 2022

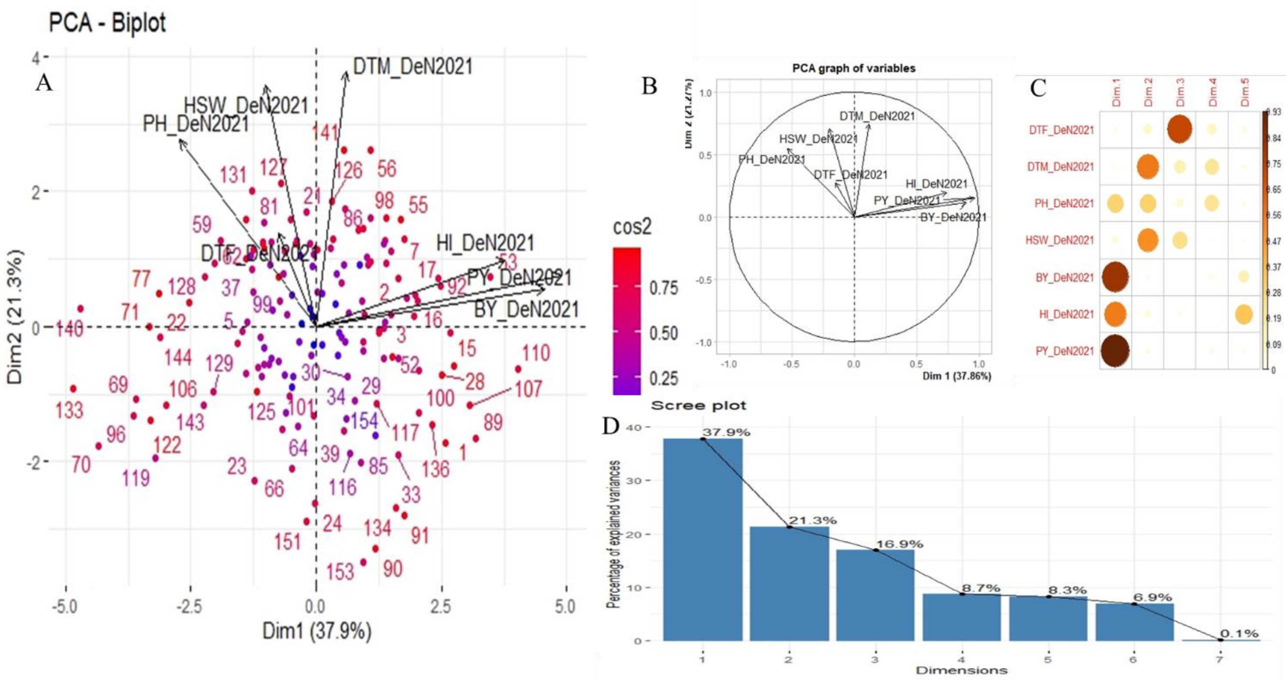

Supplementary Figure 3. Delhi 2021 timely

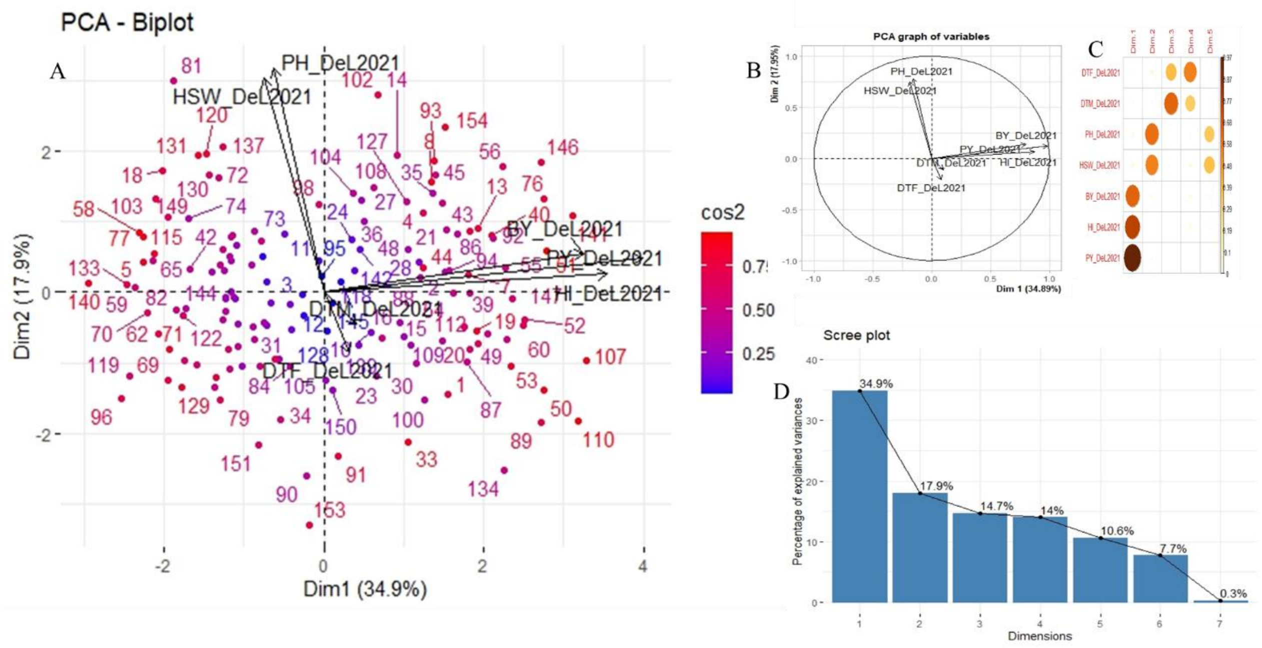

Supplementary Figure 3. Delhi 2021 late

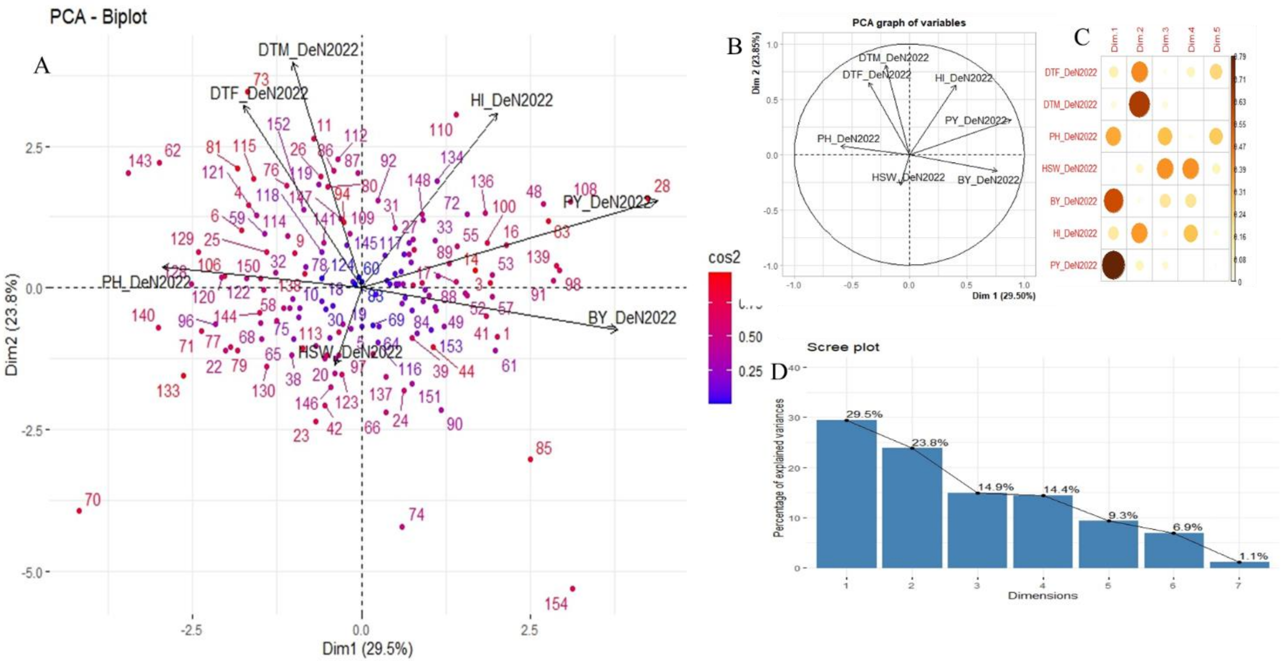

Supplementary Figure 3. Delhi 2022 timely

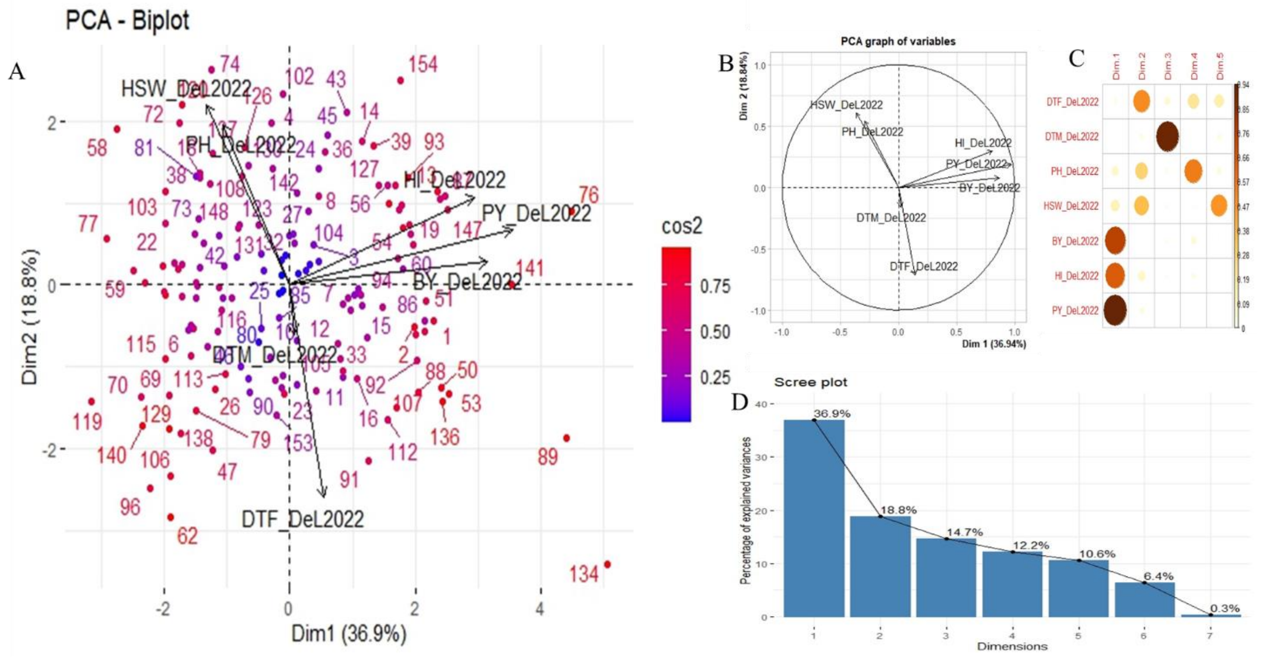

Supplementary Figure 3. Delhi 2022 late

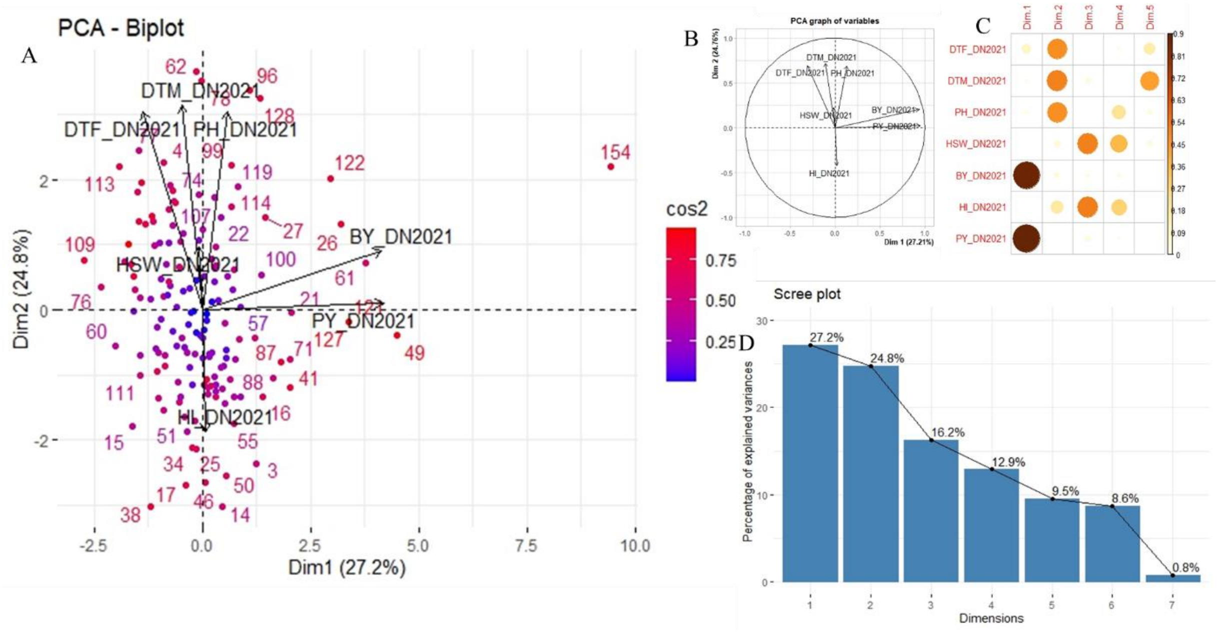

Supplementary Figure 3. Dharwad 2021 timely

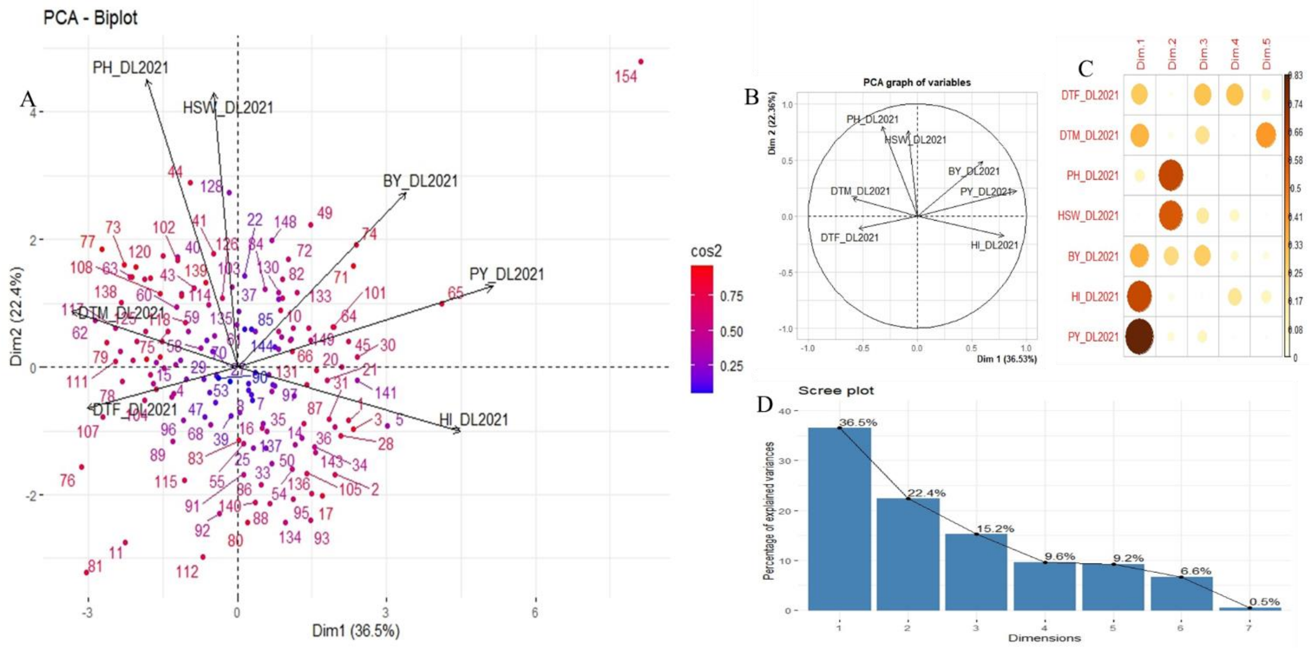

Supplementary Figure 3. Dharwad 2021 late

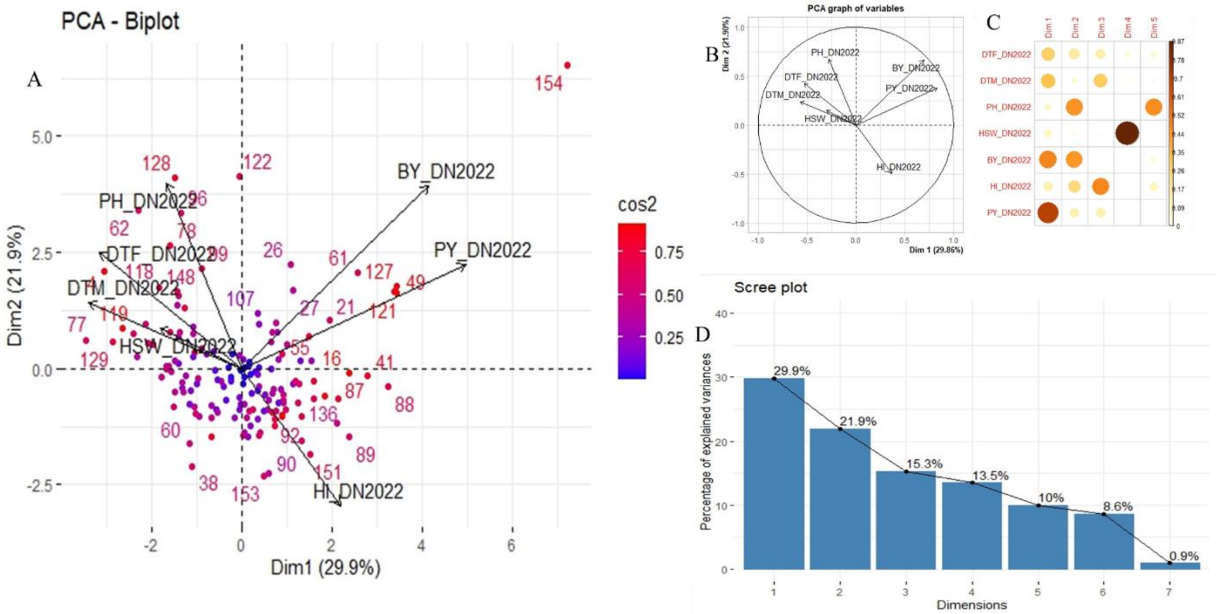

Supplementary Figure 3. Dharwad 2022 timely

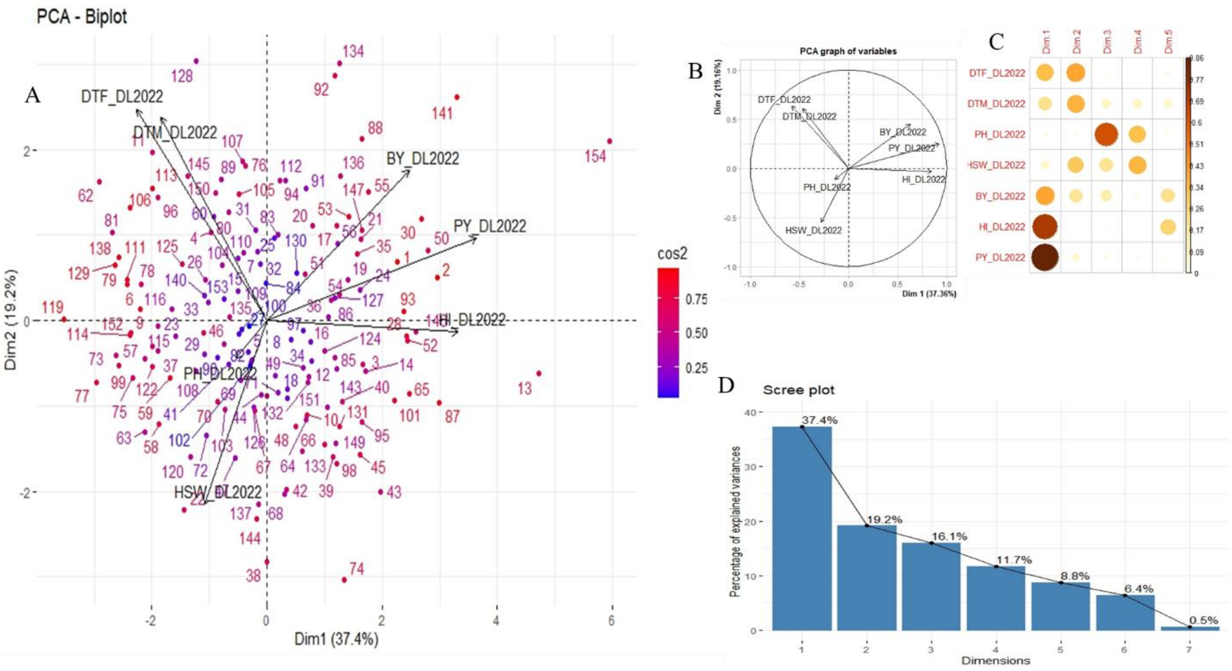

Supplementary Figure 3. Dharwad 2022 late

Figure S4a. Manhattan and respective quantile–quantile (Q–Q) plots of significant associations for studied individual traits using across location BLUPs.

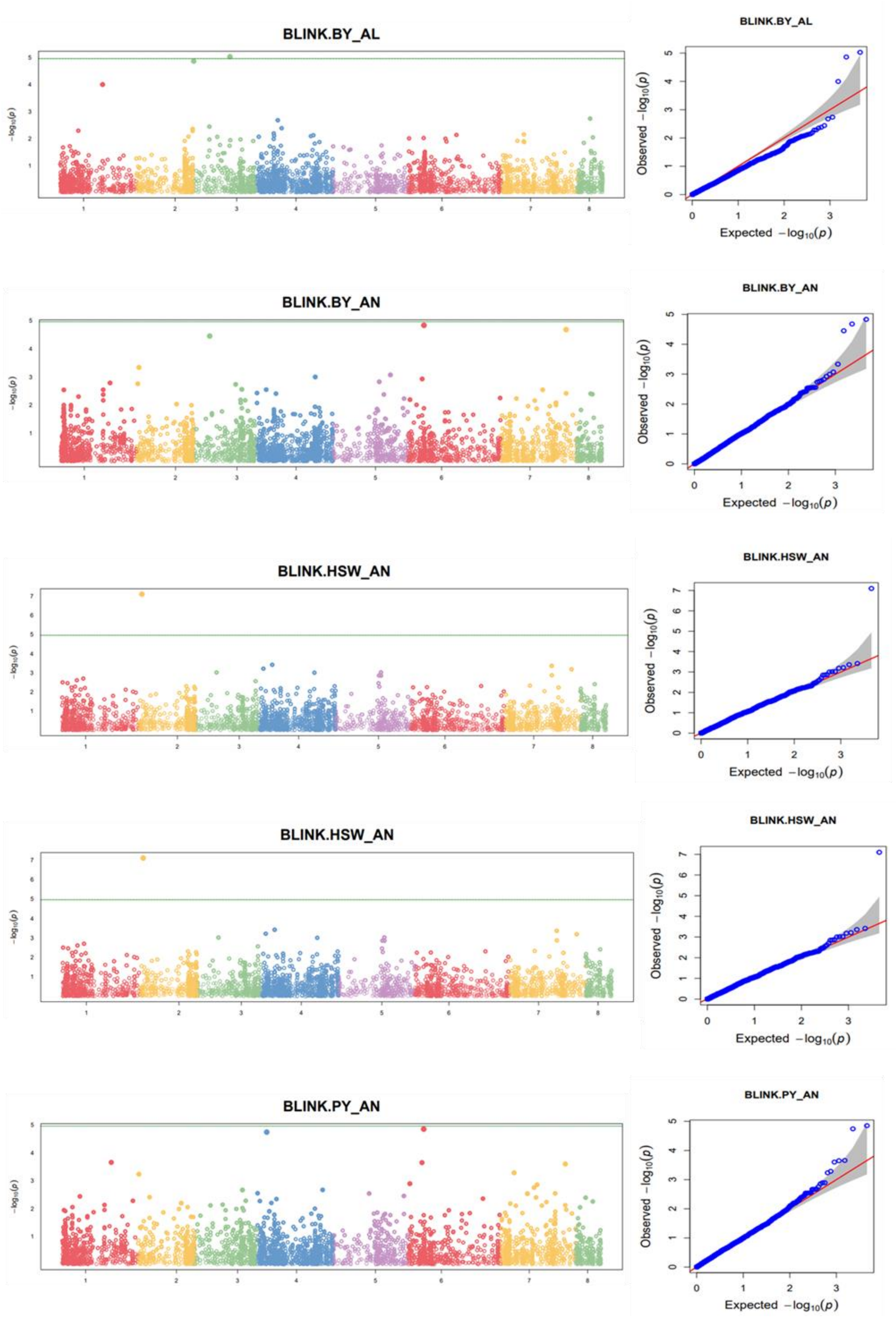

Figure S4b. Manhattan and respective quantile–quantile (Q–Q) plots of significant associations for studied individual traits using across treatment BLUPs.

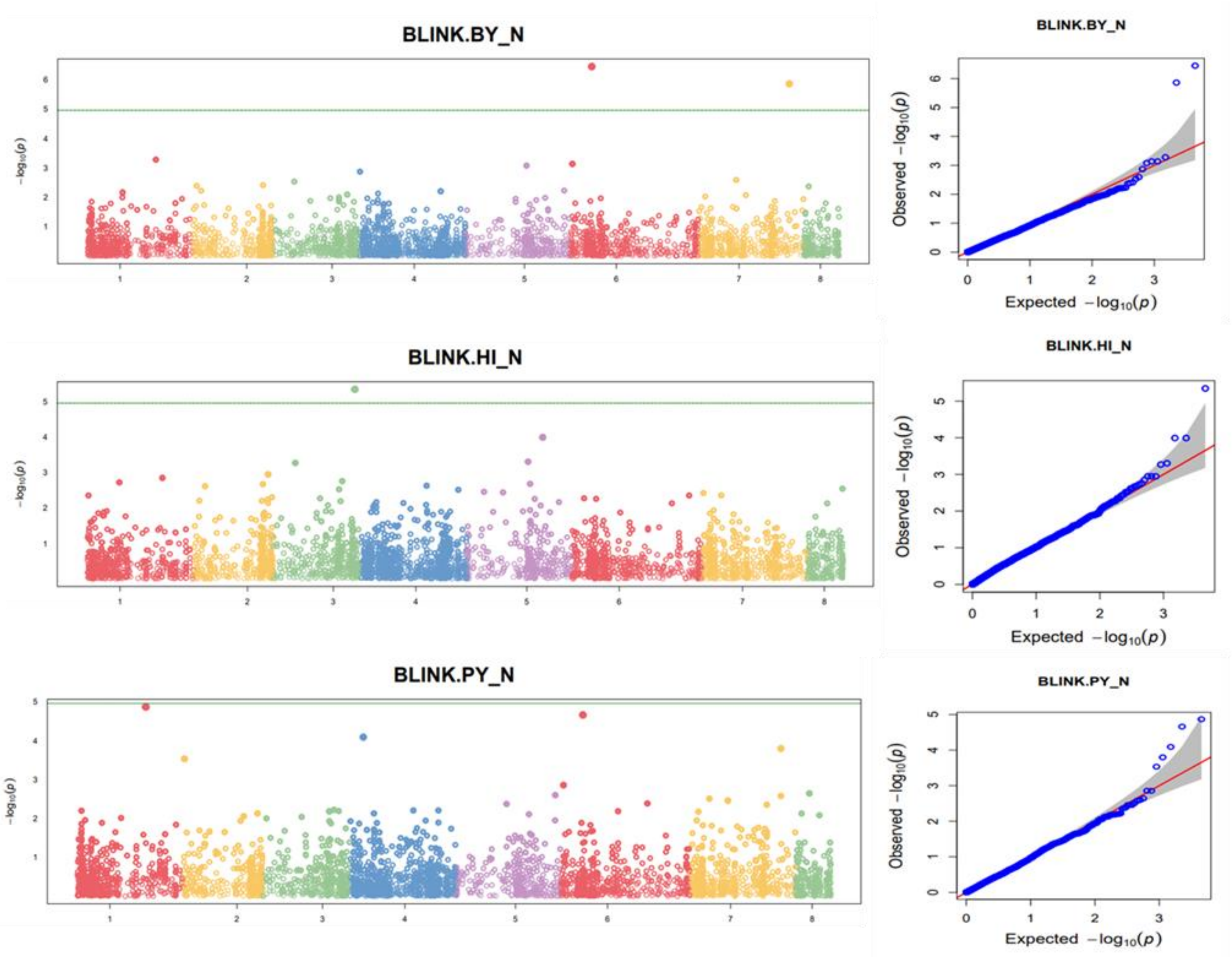

Figure S5. Significant MTAs identified on each chromosome for the studied traits.

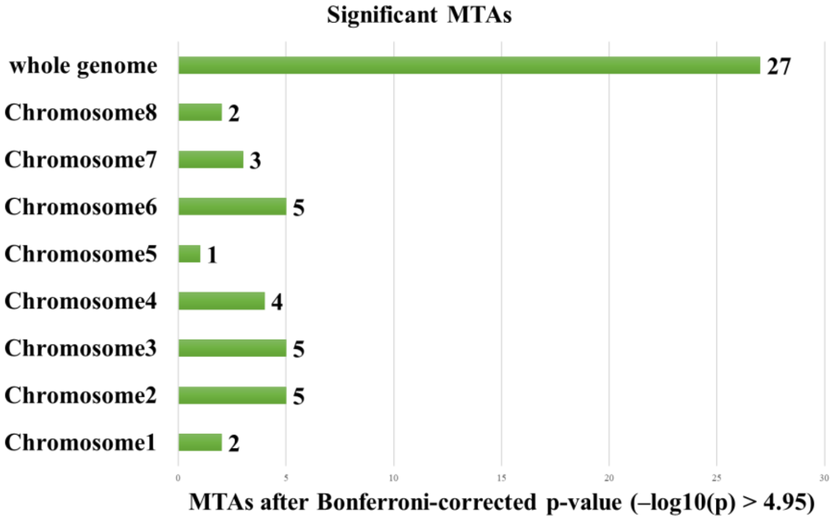

Figure S6. Allelic effects of selected MTAs identified in multiple locations for the studied traits under study.

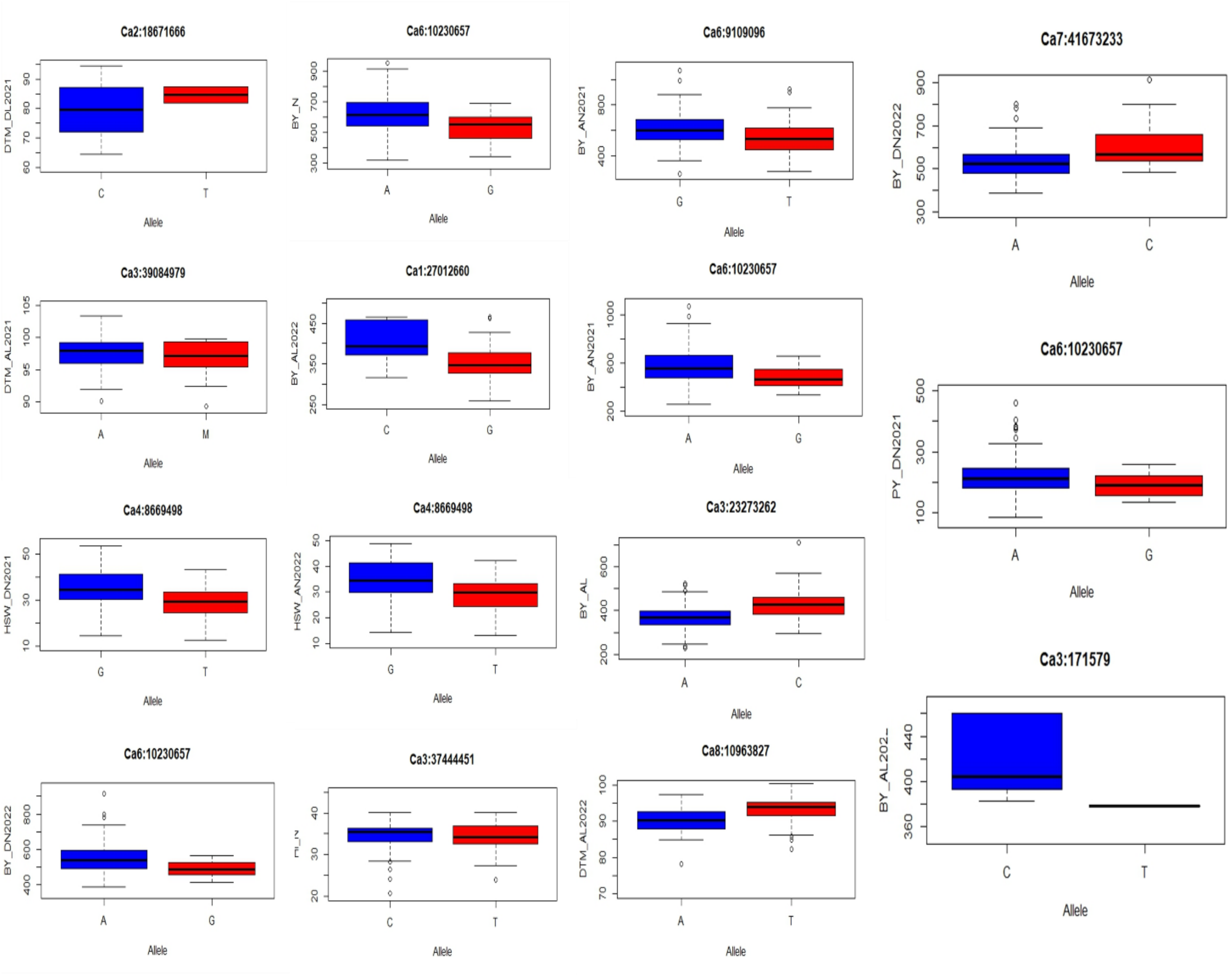

Supplement: Supplementary file 2 [file Image_1.pdf]
