# Supplementary material for: Unraveling the genetics of heat tolerance in chickpea landraces (Cicer arietinum L.) using genome-wide association studies
Source: Front Plant Sci. 2024 Mar 25;15:1376381. doi: 10.3389/fpls.2024.1376381 (PMC10999645; doi:10.3389/fpls.2024.1376381)
Supplement: Supplementary file 3 [file Table_1.docx]

**Table S1.** Details of landraces in GWAS panel, pedigree with origin

| S.No. | GENOTYPE | Accession Number | Scientific Name | IG No. | Origin | DOI |
| --- | --- | --- | --- | --- | --- | --- |
| 1 | ILC5588 | 69445 | *Cicer arietinum* | IG 69445 | FRA | 10.18730/7KWV5 |
| 2 | ILC9793 | 114759 | *Cicer arietinum* |  | AUS | 10.18730/8VVFU |
| 3 | ILC182 | 6022 | *Cicer arietinum* | IG 6022 | UKR | 10.18730/5TP1V |
| 4 | ILC5357 | 70566 | *Cicer arietinum* | IG 70566 | KGZ | 10.18730/7PKTD |
| 5 | ILC Italy | 145222 | *Cicer arietinum* | IG 145222 | ITA | 10.18730/9TSC4 |
| 6 | ILC8777 | 69767 | *Cicer arietinum* | IG 69767 | MDA | 10.18730/7M6WY |
| 7 | ILC12022 | 131610 | *Cicer arietinum* | IG 131610 | KGZ | 10.18730/9AE4N |
| 8 | ILC1932 | 7770 | *Cicer arietinum* | IG 7770 | JOR | 10.18730/5WBXC |
| 9 | ILC2377 | 8215 | *Cicer arietinum* | IG 8215 | YUG | 10.18730/5WSSC |
| 10 | ILC7274 | 74034 | *Cicer arietinum* | IG 74034 | BGR | 10.18730/7SRGG |
| 11 | ILC238 | 6076 | *Cicer arietinum* | IG 6076 | ESP | 10.18730/5TQQ7 |
| 12 | ILC6025 | 70841 | *Cicer arietinum* | IG 70841 | MEX | 10.18730/7PTYK |
| 13 | ILC11913 | 132006 | *Cicer arietinum* | IG 132006 | KEN | 10.18730/9AQR~ |
| 14 | ILC8796 | 69786 | *Cicer arietinum* | IG 69786 | CZE | 10.18730/7M7FC |
| 15 | ILC219 | 6057 | *Cicer arietinum* | IG 6057 | PAK | 10.18730/5TQ4S |
| 16 | ILC3866 | 9704 | *Cicer arietinum* | IG 9704 | BGR | 10.18730/5Y7X8 |
| 17 | ILC180 | 6018 | *Cicer arietinum* | IG 6018 | MAR | 10.18730/5TNXQ |
| 18 | ILC595 | 6433 | *Cicer arietinum* | IG 6433 | DZA | 10.18730/5V2VY |
| 19 | ILC0Australia | 0 | *Cicer arietinum* |  | Austria |  |
| 20 | ILC8797a | 69787 | *Cicer arietinum* | IG 69787 | ROU | 10.18730/7M7GD |
| 21 | ILC11902 | 131995 | *Cicer arietinum* | IG 131995 | COL | 10.18730/9AQDP |
| 22 | ILC187 | 6025 | *Cicer arietinum* | IG 6025 | UZB | 10.18730/5TP4Y |
| 23 | ILC10252 | 128273 | *Cicer arietinum* | IG 128273 | DEU | 10.18730/97AYX |
| 24 | ILC8666 | 69656 | *Cicer arietinum* | IG 69656 | PRT | 10.18730/7M3DY |
| 25 | ILC4455 | 10293 | *Cicer arietinum* | IG 10293 | ITA | 10.18730/5YSTT |
| 26 | ILC170 | 6008 | *Cicer arietinum* | IG 6008 | TUN | 10.18730/5TNME |
| 27 | ILC9856 | 125009 | *Cicer arietinum* | IG 125009 | CHN | 10.18730/94VYH |
| 28 | ILC564 | 6402 | *Cicer arietinum* | IG 6402 | LBN | 10.18730/5V1WU |
| 29 | ILC5919 | 70574 | *Cicer arietinum* | IG 70574 | RUS | 10.18730/7PM2N |
| 30 | ILC213 | 6051 | *Cicer arietinum* | IG 6051 | PER | 10.18730/5TPYK |
| 31 | ILC6891 | 73283 | *Cicer arietinum* | IG 73283 | GRC | 10.18730/7S232 |
| 32 | ILC Moldova | 145243 | *Cicer arietinum* | IG 145243 | MDA | 10.18730/9TTF2 |
| 33 | ILC10070 | 125223 | *Cicer arietinum* | IG 125223 | PSE | 10.18730/952C1 |
| 34 | ILC Romania | 145344 | *Cicer arietinum* | IG 145344 | ROU | 10.18730/9TZJH |
| 35 | ILC1934 | 7773 | *Cicer arietinum* |  | IND | 10.18730/5WC0F |
| 36 | ILC593 | 6431 | *Cicer arietinum* | IG 6431 | FRA | 10.18730/5V2SW |
| 37 | ILC8632 | 69622 | *Cicer arietinum* | IG 69622 | BGD | 10.18730/7M2B~ |
| 38 | ILC239 | 6077 | *Cicer arietinum* | IG 6077 | ETH | 10.18730/5TQR8 |
| 39 | ILC3887 | 9725 | *Cicer arietinum* | IG 9725 | CHL | 10.18730/5Y8JX |
| 40 | ILC1272 | 7110 | *Cicer arietinum* |  | TUR | 10.18730/5VQX1 |
| 41 | ILC6062 | 70878 | *Cicer arietinum* | IG 70878 | CHL | 10.18730/7PW2J |
| 42 | ILC234 | 6072 | *Cicer arietinum* | IG 6072 | IRQ | 10.18730/5TQK3 |
| 43 | ILC1312 | 7150 | *Cicer arietinum* | IG 7150 | CYP | 10.18730/5VS43 |
| 44 | ILC4432a | 10270 | *Cicer arietinum* | IG 10270 | ESP | 10.18730/5YS33 |
| 45 | ILC4432b | 10270 | *Cicer arietinum* | IG 10270 | ESP | 10.18730/5YS33 |
| 46 | ILC7187 | 73579 | *Cicer arietinum* |  | --- | 10.18730/7SB5~ |
| 47 | ILC184 | 6022 | *Cicer arietinum* | IG 6022 | UKR | 10.18730/5TP1V |
| 48 | ILC5595 | 70250 | *Cicer arietinum* | IG 70250 | PAK | 10.18730/7MMNV |
| 49 | ILC445 | 6283 | *Cicer arietinum* | IG 6283 | USA | 10.18730/5TY5W |
| 50 | ILC Russia | 145171 | *Cicer arietinum* | IG 145171 | RUS | 10.18730/9TPV$ |
| 51 | ILC8797b | 69787 | *Cicer arietinum* | IG 69787 | ROU | 10.18730/7M7GD |
| 52 | ILC228 | 6066 | *Cicer arietinum* | IG 6066 | TUR | 10.18730/5TQD$ |
| 53 | ILC8730 | 69720 | *Cicer arietinum* | IG 69720 | USA | 10.18730/7M5DM |
| 54 | ILC211 | 6049 | *Cicer arietinum* | IG 6049 | IND | 10.18730/5TPWH |
| 55 | ILC1313 | 7151 | *Cicer arietinum* | IG 7151 | ETH | 10.18730/5VS54 |
| 56 | ILC1946 | 7784 | *Cicer arietinum* |  | IND | 10.18730/5WCBT |
| 57 | ILC1906 | 7744 | *Cicer arietinum* | IG 7744 | SYR | 10.18730/5WB4R |
| 58 | ILC5911 | 70566 | *Cicer arietinum* | IG 70566 | KGZ | 10.18730/7PKTD |
| 59 | ILC167 | 6005 | *Cicer arietinum* |  | TUN | 10.18730/5TNHB |
| 60 | ILC35 | 5873 | *Cicer arietinum* |  | SYR | 10.18730/5THFX |
| 61 | ILC160 | 5998 | *Cicer arietinum* |  | TUN | 10.18730/5TNA4 |
| 62 | ILC139 | 5977 | *Cicer arietinum* |  | ESP | 10.18730/5TMQP |
| 63 | ILC157 | 5995 | *Cicer arietinum* |  | TUN | 10.18730/5TN71 |
| 64 | IG5839 | 5839 | *Cicer arietinum* |  | JOR | 10.18730/5TGD* |
| 65 | IG5842 | 5842 | *Cicer arietinum* |  | JOR | 10.18730/5TGG= |
| 66 | IG5843 | 5843 | *Cicer arietinum* |  | JOR | 10.18730/5TGHU |
| 67 | IG5844a | 5844 | *Cicer arietinum* |  | JOR | 10.18730/5TGJ0 |
| 68 | IG5844b | 5844 | *Cicer arietinum* |  | JOR | 10.18730/5TGJ0 |
| 69 | IG5845 | 5845 | *Cicer arietinum* |  | JOR | 10.18730/5TGK1 |
| 70 | IG5846 | 5846 | *Cicer arietinum* |  | JOR | 10.18730/5TGM2 |
| 71 | IG5847 | 5847 | *Cicer arietinum* |  | JOR | 10.18730/5TGN3 |
| 72 | IG5848 | 5848 | *Cicer arietinum* |  | JOR | 10.18730/5TGP4 |
| 73 | IG5849 | 5849 | *Cicer arietinum* |  | JOR | 10.18730/5TGQ5 |
| 74 | IG5850 | 5850 | *Cicer arietinum* |  | JOR | 10.18730/5TGR6 |
| 75 | IG5851a | 5851 | *Cicer arietinum* |  | JOR | 10.18730/5TGS7 |
| 76 | IG5851b | 5851 | *Cicer arietinum* |  | JOR | 10.18730/5TGS7 |
| 77 | IG5852 | 5852 | *Cicer arietinum* |  | JOR | 10.18730/5TGT8 |
| 78 | IG5853 | 5853 | *Cicer arietinum* |  | JOR | 10.18730/5TGV9 |
| 79 | IG5854 | 5854 | *Cicer arietinum* |  | JOR | 10.18730/5TGWA |
| 80 | IG5855 | 5855 | *Cicer arietinum* |  | JOR | 10.18730/5TGXB |
| 81 | IG5856 | 5856 | *Cicer arietinum* |  | JOR | 10.18730/5TGYC |
| 82 | IG5857 | 5857 | *Cicer arietinum* |  | JOR | 10.18730/5TGZD |
| 83 | IG5858 | 5858 | *Cicer arietinum* |  | JOR | 10.18730/5TH0E |
| 84 | IG5859 | 5859 | *Cicer arietinum* |  | JOR | 10.18730/5TH1F |
| 85 | IG5860 | 5860 | *Cicer arietinum* |  | JOR | 10.18730/5TH2G |
| 86 | IG5861 | 5861 | *Cicer arietinum* |  | JOR | 10.18730/5TH3H |
| 87 | IG5862 | 5862 | *Cicer arietinum* |  | JOR | 10.18730/5TH4J |
| 88 | IG5863 | 5863 | *Cicer arietinum* |  | JOR | 10.18730/5TH5K |
| 89 | IG5864 | 5864 | *Cicer arietinum* |  | JOR | 10.18730/5TH6M |
| 90 | IG5865 | 5865 | *Cicer arietinum* |  | JOR | 10.18730/5TH7N |
| 91 | IG5866 | 5866 | *Cicer arietinum* |  | JOR | 10.18730/5TH8P |
| 92 | IG5867 | 5867 | *Cicer arietinum* |  | JOR | 10.18730/5TH9Q |
| 93 | IG5868 | 5868 | *Cicer arietinum* |  | SYR | 10.18730/5THAR |
| 94 | IG5869 | 5869 | *Cicer arietinum* |  | SYR | 10.18730/5THBS |
| 95 | IG5870 | 5870 | *Cicer arietinum* |  | SYR | 10.18730/5THCT |
| 96 | IG5871 | 5871 | *Cicer arietinum* |  | SYR | 10.18730/5THDV |
| 97 | IG5872 | 5872 | *Cicer arietinum* |  | SYR | 10.18730/5THEW |
| 98 | IG5874 | 5874 | *Cicer arietinum* |  | SYR | 10.18730/5THGY |
| 99 | IG5875 | 5875 | *Cicer arietinum* |  | SYR | 10.18730/5THHZ |
| 100 | IG5876 | 5876 | *Cicer arietinum* |  | SYR | 10.18730/5THJ* |
| 101 | IG5877 | 5877 | *Cicer arietinum* |  | SYR | 10.18730/5THK~ |
| 102 | IG5878 | 5878 | *Cicer arietinum* |  | SYR | 10.18730/5THM$ |
| 103 | IG5879 | 5879 | *Cicer arietinum* |  | SYR | 10.18730/5THN= |
| 104 | IG5880 | 5880 | *Cicer arietinum* |  | SYR | 10.18730/5THPU |
| 105 | IG5881 | 5881 | *Cicer arietinum* |  | SYR | 10.18730/5THQ0 |
| 106 | IG5882 | 5882 | *Cicer arietinum* |  | SYR | 10.18730/5THR1 |
| 107 | IG5883 | 5883 | *Cicer arietinum* |  | IRQ | 10.18730/5THS2 |
| 108 | IG5884 | 5884 | *Cicer arietinum* |  | IRQ | 10.18730/5THT3 |
| 109 | IG5885 | 5885 | *Cicer arietinum* |  | IRQ | 10.18730/5THV4 |
| 110 | IG5886 | 5886 | *Cicer arietinum* |  | IRQ | 10.18730/5THW5 |
| 111 | IG5888 | 5888 | *Cicer arietinum* |  | IRQ | 10.18730/5THY7 |
| 112 | IG5889 | 5889 | *Cicer arietinum* |  | IRQ | 10.18730/5THZ8 |
| 113 | IG5890 | 5890 | *Cicer arietinum* |  | IRQ | 10.18730/5TJ09 |
| 114 | IG5891 | 5891 | *Cicer arietinum* |  | IRQ | 10.18730/5TJ1A |
| 115 | IG5892 | 5892 | *Cicer arietinum* |  | IRQ | 10.18730/5TJ2B |
| 116 | IG5893 | 5893 | *Cicer arietinum* |  | IRQ | 10.18730/5TJ3C |
| 117 | IG5894 | 5894 | *Cicer arietinum* |  | IRQ | 10.18730/5TJ4D |
| 118 | IG5895 | 5895 | *Cicer arietinum* |  | IRQ | 10.18730/5TJ5E |
| 119 | IG5896 | 5896 | *Cicer arietinum* |  | IRQ | 10.18730/5TJ6F |
| 120 | IG5897 | 5897 | *Cicer arietinum* |  | IRQ | 10.18730/5TJ7G |
| 121 | IG5898 | 5898 | *Cicer arietinum* |  | IRQ | 10.18730/5TJ8H |
| 122 | IG5899 | 5899 | *Cicer arietinum* |  | IRQ | 10.18730/5TJ9J |
| 123 | IG5900 | 5900 | *Cicer arietinum* |  | IRQ | 10.18730/5TJAK |
| 124 | IG5901 | 5901 | *Cicer arietinum* |  | IRQ | 10.18730/5TJBM |
| 125 | IG5902 | 5902 | *Cicer arietinum* |  | IRQ | 10.18730/5TJCN |
| 126 | IG5903 | 5903 | *Cicer arietinum* |  | IRQ | 10.18730/5TJDP |
| 127 | IG5904 | 5904 | *Cicer arietinum* |  | IRQ | 10.18730/5TJEQ |
| 128 | IG5905 | 5905 | *Cicer arietinum* |  | IRQ | 10.18730/5TJFR |
| 129 | IG5906 | 5906 | *Cicer arietinum* |  | IRQ | 10.18730/5TJGS |
| 130 | IG5907 | 5907 | *Cicer arietinum* |  | IRQ | 10.18730/5TJHT |
| 131 | IG5908 | 5908 | *Cicer arietinum* |  | IRQ | 10.18730/5TJJV |
| 132 | IG5909 | 5909 | *Cicer arietinum* |  | IRQ | 10.18730/5TJKW |
| 133 | IG5978 | 5978 | *Cicer arietinum* |  | ESP | 10.18730/5TMRQ |
| 134 | IG5980 | 5980 | *Cicer arietinum* |  | ESP | 10.18730/5TMSR |
| 135 | IG5982 | 5982 | *Cicer arietinum* |  | ESP | 10.18730/5TMTS |
| 136 | IG5983 | 5984 | *Cicer arietinum* |  | ESP | 10.18730/5TMWV |
| 137 | IG5984 | 5984 | *Cicer arietinum* |  | ESP | 10.18730/5TMWV |
| 138 | IG5985 | 5985 | *Cicer arietinum* |  | ESP | 10.18730/5TMXW |
| 139 | IG5986 | 5986 | *Cicer arietinum* |  | ESP | 10.18730/5TMYX |
| 140 | IG5987 | 5987 | *Cicer arietinum* |  | ESP | 10.18730/5TMZY |
| 141 | IG5988 | 5988 | *Cicer arietinum* |  | ESP | 10.18730/5TN0Z |
| 142 | IG5990 | 5991 | *Cicer arietinum* |  | GRC | 10.18730/5TN3$ |
| 143 | IG5991 | 5991 | *Cicer arietinum* |  | GRC | 10.18730/5TN3$ |
| 144 | IG5992 | 5992 | *Cicer arietinum* |  | GRC | 10.18730/5TN4= |
| 145 | IG5993 | 5993 | *Cicer arietinum* |  | GRC | 10.18730/5TN5U |
| 146 | IG5997 | 5997 | *Cicer arietinum* |  | TUN | 10.18730/5TN93 |
| 147 | IG5999 | 5999 | *Cicer arietinum* |  | TUN | 10.18730/5TNB5 |
| 148 | IG6000 | 6000 | *Cicer arietinum* |  | TUN | 10.18730/5TNC6 |
| 149 | IG6001 | 6001 | *Cicer arietinum* |  | TUN | 10.18730/5TND7 |
| 150 | IG6002 | 6003 | *Cicer arietinum* |  | TUN | 10.18730/5TNF9 |
| 151 | IG6003 | 6003 | *Cicer arietinum* |  | TUN | 10.18730/5TNF9 |
| 152 | ILC8758 | 114804 | *Cicer arietinum* | IG 114804 | ECU | 10.18730/8VWQ2 |
| 153 | IG6006 | 6006 | *Cicer arietinum* |  | TUN | 10.18730/5TNJC |
| 154 | JG14 | 92944 | *Cicer arietinum* | 92944 | Jabalpur, India | ICC 92944 |

**Table S2.** Descriptive statistics and ANOVA of all the studied traits under individual location and year.

| S.No. | Traits | Max | Min | GM | (SEm) | (CD) 1% | EV | GV | PV | ECV | GCV | PCV | H2 | GA | GA% Mean | G Mean sq |
| --- | --- | --- | --- | --- | --- | --- | --- | --- | --- | --- | --- | --- | --- | --- | --- | --- |
| 1 | BY_AL2021 | 1125 | 83.07 | 377.5 | 45.53 | 166.91 | 6220.22 | 7100.19 | 13320.41 | 20.89 | 22.32 | 30.57 | 0.53 | 126.73 | 33.57 | 27521*** |
| 2 | BY_AL2022 | 1151.3 | 99.53 | 361.2177 | 60.2316 | 220.7866 | 10883.53 | 5111.329 | 15994.86 | 28.8812 | 19.7924 | 35.0123 | 0.3196 | 83.2551 | 23.0485 | 26218*** |
| 3 | BY_AN2021 | 3121.3 | 140.39 | 594.36 | 85.24 | 312.47 | 21799.49 | 73442.84 | 95242.34 | 24.84 | 45.6 | 51.92 | 0.77 | 490.23 | 82.48 | 242128*** |
| 4 | BY_AN2022 | 3246.24 | 160 | 695.0582 | 147.0832 | 539.1524 | 64900.39 | 75199.99 | 140100.4 | 36.6524 | 39.4537 | 53.8516 | 0.5368 | 413.8713 | 59.5448 | 290500*** |
| 5 | BY_DeL2021 | 838 | 195 | 437.19 | 54.42 | 199.5 | 8885.64 | 9352.81 | 18238.45 | 21.56 | 22.12 | 30.89 | 0.51 | 142.66 | 32.63 | 36944*** |
| 6 | BY_DeL2022 | 860.48 | 170.83 | 428.7743 | 67.679 | 248.0861 | 13741.33 | 7418.074 | 21159.41 | 27.3392 | 20.0871 | 33.9252 | 0.3506 | 105.0526 | 24.5007 | 35996*** |
| 7 | BY_DeN2021 | 1079 | 180 | 550.58 | 50.35 | 184.57 | 7605.84 | 19692.54 | 27298.38 | 15.84 | 25.49 | 30.01 | 0.72 | 245.53 | 44.59 | 66683*** |
| 8 | BY_DeN2022 | 3040 | 160 | 702.7702 | 165.5142 | 606.7137 | 82184.86 | 16532.24 | 98717.1 | 40.7927 | 18.2958 | 44.7078 | 0.1675 | 108.3934 | 15.4237 | 131782*** |
| 9 | BY_DL2021 | 1151.3 | 97.26 | 353.58 | 56.7 | 207.83 | 9644.04 | 8779.72 | 18423.76 | 27.77 | 26.5 | 38.39 | 0.48 | 133.25 | 37.69 | 35983*** |
| 10 | BY_DL2022 | 995.44 | 83.07 | 383.3039 | 66.9099 | 245.2668 | 13430.8 | 2050.078 | 15480.88 | 30.2348 | 11.8125 | 32.4605 | 0.1324 | 33.9422 | 8.8552 | 19581*** |
| 11 | BY_DN2021 | 3246.24 | 140.39 | 630.46 | 85.28 | 312.62 | 21819.83 | 76340.1 | 98159.92 | 23.43 | 43.82 | 49.69 | 0.78 | 501.94 | 79.61 | 250840*** |
| 12 | BY_DN2022 | 3080.93 | 140.39 | 547.5955 | 122.9807 | 450.8017 | 45372.77 | 23704.99 | 69077.76 | 38.8989 | 28.1164 | 47.9965 | 0.3432 | 185.7966 | 33.9295 | 116488*** |
| 13 | DTF_AL2021 | 79 | 30 | 51.13 | 2.35 | 8.61 | 16.55 | 25.52 | 42.07 | 7.96 | 9.88 | 12.68 | 0.61 | 8.11 | 15.85 | 93.11*** |
| 14 | DTF_AL2022 | 75 | 35 | 50.0065 | 2.6347 | 9.6578 | 20.8247 | 16.1833 | 37.008 | 9.1256 | 8.0446 | 12.1653 | 0.4373 | 5.4801 | 10.9588 | 69.37*** |
| 15 | DTF_AN2021 | 127 | 48 | 76.85 | 6.11 | 22.38 | 111.81 | 113.56 | 225.37 | 13.76 | 13.87 | 19.54 | 0.5 | 15.58 | 20.28 | 452.5*** |
| 16 | DTF_AN2022 | 102 | 47 | 72.3831 | 3.9388 | 14.4381 | 46.5421 | 43.0303 | 89.5724 | 9.4251 | 9.0625 | 13.0752 | 0.4804 | 9.366 | 12.9395 | 175.63*** |
| 17 | DTF_DeL2021 | 79 | 39 | 54.17 | 1.51 | 5.52 | 6.81 | 21 | 27.81 | 4.82 | 8.46 | 9.73 | 0.76 | 8.2 | 15.14 | 69.8*** |
| 18 | DTF_DeL2022 | 79 | 27 | 57.803 | 2.7512 | 10.0847 | 22.7066 | 7.745 | 30.4516 | 8.2438 | 4.8146 | 9.5467 | 0.2543 | 2.8912 | 5.0018 | 45.9*** |
| 19 | DTF_DeN2021 | 111 | 48 | 83.49 | 2.41 | 8.82 | 17.37 | 8.75 | 26.12 | 4.99 | 3.54 | 6.12 | 0.33 | 3.53 | 4.22 | 43.6*** |
| 20 | DTF_DeN2022 | 111 | 52 | 84.0584 | 2.9124 | 10.6757 | 25.4458 | 9.2307 | 34.6765 | 6.001 | 3.6144 | 7.0055 | 0.2662 | 3.2291 | 3.8415 | 53.1*** |
| 21 | DTF_DL2021 | 75 | 30 | 48.34 | 2.91 | 10.67 | 25.43 | 13.03 | 38.46 | 10.43 | 7.47 | 12.83 | 0.34 | 4.33 | 8.95 | 64.52*** |
| 22 | DTF_DL2022 | 67 | 30 | 50.645 | 1.9102 | 7.002 | 10.9464 | 18.1982 | 29.1446 | 6.5328 | 8.4232 | 10.6596 | 0.6244 | 6.9441 | 13.7113 | 65.54*** |
| 23 | DTF_DN2021 | 102 | 47 | 70.61 | 3.76 | 13.77 | 42.32 | 51.25 | 93.57 | 9.21 | 10.14 | 13.7 | 0.55 | 10.91 | 15.46 | 196.07*** |
| 24 | DTF_DN2022 | 125 | 48 | 76.3853 | 5.1216 | 18.7738 | 78.6914 | 39.4371 | 118.1285 | 11.6133 | 8.2213 | 14.2288 | 0.3338 | 7.4747 | 9.7855 | 197*** |
| 25 | DTM_AL2021 | 121 | 55 | 97.13 | 2.94 | 10.77 | 25.89 | 15.52 | 41.4 | 5.24 | 4.06 | 6.62 | 0.37 | 4.97 | 5.11 | 72.43*** |
| 26 | DTM_AL2022 | 113 | 59 | 92.7056 | 2.1306 | 7.81 | 13.6185 | 20.8969 | 34.5154 | 3.9807 | 4.931 | 6.3372 | 0.6054 | 7.3273 | 7.9038 | 76.31*** |
| 27 | DTM_AN2021 | 131 | 108 | 122.48 | 1.59 | 5.85 | 7.63 | 14.41 | 22.04 | 2.26 | 3.1 | 3.83 | 0.65 | 6.32 | 5.16 | 50.871*** |
| 28 | DTM_AN2022 | 145 | 75 | 117.6818 | 3.4761 | 12.7419 | 36.2489 | 37.5399 | 73.7888 | 5.1161 | 5.2064 | 7.2994 | 0.5087 | 9.0025 | 7.6499 | 148.9*** |
| 29 | DTM_DeL2021 | 109 | 71 | 87.22 | 0.78 | 2.87 | 1.84 | 14.09 | 15.93 | 1.56 | 4.3 | 4.58 | 0.88 | 7.27 | 8.34 | 44.1*** |
| 30 | DTM_DeL2022 | 113 | 50 | 90.7944 | 1.7234 | 6.3172 | 8.91 | 14.9228 | 23.8328 | 3.2876 | 4.2547 | 5.3769 | 0.6261 | 6.2969 | 6.9353 | 53.68*** |
| 31 | DTM_DeN2021 | 159 | 111 | 135.98 | 2.83 | 10.36 | 23.95 | 3.05 | 27 | 3.6 | 1.29 | 3.82 | 0.11 | 1.21 | 0.89 | 33.1*** |
| 32 | DTM_DeN2022 | 159 | 110 | 136.1126 | 2.5223 | 9.2458 | 19.0859 | 6.5113 | 25.5972 | 3.2097 | 1.8747 | 3.717 | 0.2544 | 2.6512 | 1.9478 | 38.6*** |
| 33 | DTM_DL2021 | 104 | 59 | 91.11 | 0.98 | 3.58 | 2.86 | 40.2 | 43.06 | 1.86 | 6.96 | 7.2 | 0.93 | 12.62 | 13.85 | 123.45*** |
| 34 | DTM_DL2022 | 110 | 57 | 94.4221 | 2.4446 | 8.9609 | 17.9277 | 8.9944 | 26.9221 | 4.4842 | 3.1762 | 5.4952 | 0.3341 | 3.571 | 3.782 | 44.91*** |
| 35 | DTM_DN2021 | 135 | 75 | 115.05 | 1.26 | 4.6 | 4.73 | 82.46 | 87.2 | 1.89 | 7.89 | 8.12 | 0.95 | 18.19 | 15.81 | 252.12*** |
| 36 | DTM_DN2022 | 143 | 108 | 127.171 | 2.011 | 7.3714 | 12.1319 | 6.2934 | 18.4253 | 2.7389 | 1.9727 | 3.3754 | 0.3416 | 3.0203 | 2.375 | 31*** |
| 37 | HI_AL2021 | 45.97 | 1.97 | 24.59 | 3.45 | 12.66 | 35.8 | 72.14 | 107.94 | 24.33 | 34.54 | 42.25 | 0.67 | 14.3 | 58.17 | 252.23*** |
| 38 | HI_AL2022 | 42.72 | 0.64 | 25.2553 | 3.5543 | 13.0287 | 37.8991 | 68.7466 | 106.6457 | 24.376 | 32.8302 | 40.8903 | 0.6446 | 13.7135 | 54.2996 | 244.1*** |
| 39 | HI_AN2021 | 47.87 | 8.62 | 36.09 | 2.91 | 10.65 | 25.34 | 13.15 | 38.48 | 13.95 | 10.05 | 17.19 | 0.34 | 4.37 | 12.1 | 64.77*** |
| 40 | HI_AN2022 | 43.98 | 3.15 | 33.5751 | 3.6486 | 13.3745 | 39.9374 | 10.3871 | 50.3245 | 18.8223 | 9.5991 | 21.1287 | 0.2064 | 3.0163 | 8.9837 | 71.1*** |
| 41 | HI_DeL2021 | 44.57 | 3.9 | 29.7145 | 2.9063 | 10.6534 | 25.3397 | 86.653 | 111.9927 | 16.9407 | 31.3273 | 35.6144 | 0.7737 | 16.8677 | 56.7658 | 285.3*** |
| 42 | HI_DeL2022 | 44.09 | 2.71 | 29.4205 | 5.0802 | 18.622 | 77.4242 | 31.4098 | 108.834 | 29.9081 | 19.0495 | 35.4595 | 0.2886 | 6.2023 | 21.0816 | 171.7*** |
| 43 | HI_DeN2021 | 45.35 | 3.15 | 35.19 | 3.17 | 11.63 | 30.21 | 20.02 | 50.23 | 15.62 | 12.71 | 20.14 | 0.4 | 5.82 | 16.54 | 90.28*** |
| 44 | HI_DeN2022 | 45.18 | 3.15 | 32.6534 | 4.8687 | 17.8469 NS | 71.1128 | 5.9775 | 77.0903 | 25.8253 | 7.4874 | 26.8888 | 0.0775 | 1.4024 | 4.2948 | 89 |
| 45 | HI_DL2021 | 45.6 | 3.16 | 28.45 | 3.41 | 12.51 | 34.96 | 67.95 | 102.91 | 20.78 | 28.98 | 35.66 | 0.66 | 13.8 | 48.5 | 238.82*** |
| 46 | HI_DL2022 | 44.09 | 1.97 | 25.9913 | 5.407 | 19.8202 | 87.708 | 26.9713 | 114.6793 | 36.0323 | 19.9813 | 41.2017 | 0.2352 | 5.1883 | 19.9617 | 168.6*** |
| 47 | HI_DN2021 | 46.93 | 12.54 | 34.85 | 2.84 | 10.41 | 24.18 | 12.89 | 37.06 | 14.11 | 10.3 | 17.47 | 0.35 | 4.36 | 12.51 | 62.833*** |
| 48 | HI_DN2022 | 46.72 | 3.15 | 35.1635 | 3.8925 | 14.2685 NS | 45.4551 | 5.4174 | 50.8725 | 19.1734 | 6.6192 | 20.2838 | 0.1065 | 1.5646 | 4.4495 | 61.7*** |
| 49 | HSW_AL2021 | 57.94 | 8.41 | 27.45 | 1.91 | 7.01 | 10.98 | 50.81 | 61.79 | 12.07 | 25.96 | 28.63 | 0.82 | 13.32 | 48.5 | 163.42*** |
| 50 | HSW_AL2022 | 47.61 | 5.86 | 27.353 | 2.0091 | 7.3646 | 12.1095 | 53.9297 | 66.0392 | 12.7221 | 26.8478 | 29.7095 | 0.8166 | 13.6708 | 49.9791 | 173.89*** |
| 51 | HSW_AN2021 | 56.8 | 9.46 | 30.25 | 2.15 | 7.87 | 13.82 | 58.93 | 72.75 | 12.29 | 25.37 | 28.19 | 0.81 | 14.23 | 47.04 | 190.61*** |
| 52 | HSW_AN2022 | 57.97 | 5.86 | 30.9274 | 2.597 | 9.5197 | 20.2336 | 59.7792 | 80.0128 | 14.5443 | 24.9995 | 28.9225 | 0.7471 | 13.7669 | 44.5136 | 199.57*** |
| 53 | HSW_DeL2021 | 20.02 | 2.68 | 11.62 | 0.93 | 3.42 | 2.62 | 8.46 | 11.08 | 13.93 | 25.04 | 28.65 | 0.76 | 5.24 | 45.08 | 28.01*** |
| 54 | HSW_DeL2022 | 43.52 | 3 | 16.1248 | 2.5974 | 9.5213 | 20.2402 | 7.9037 | 28.1439 | 27.9005 | 17.4349 | 32.9 | 0.2808 | 3.0691 | 19.0334 | 44*** |
| 55 | HSW_DeN2021 | 22.8 | 1.12 | 12.77 | 1.67 | 6.12 | 8.37 | 9.64 | 18.01 | 22.65 | 24.31 | 33.22 | 0.54 | 4.68 | 36.63 | 37.28*** |
| 56 | HSW_DeN2022 | 55 | 1.12 | 18.0406 | 2.9219 | 10.7107 | 25.6131 | 12.5766 | 38.1897 | 28.053 | 19.6576 | 34.2548 | 0.3293 | 4.1923 | 23.2381 | 63.3*** |
| 57 | HSW_DL2021 | 47.61 | 8.52 | 27.21 | 2.29 | 8.38 | 15.67 | 49.87 | 65.54 | 14.55 | 25.95 | 29.75 | 0.76 | 12.69 | 46.63 | 165.28*** |
| 58 | HSW_DL2022 | 43.75 | 4.12 | 21.5631 | 2.6674 | 9.7776 | 21.3449 | 20.1384 | 41.4833 | 21.4257 | 20.8114 | 29.8693 | 0.4855 | 6.441 | 29.8704 | 81.8*** |
| 59 | HSW_DN2021 | 57.97 | 10.33 | 31.08 | 2.56 | 9.38 | 19.66 | 61.05 | 80.71 | 14.27 | 25.14 | 28.9 | 0.76 | 14 | 45.04 | 202.8*** |
| 60 | HSW_DN2022 | 55.92 | 4.12 | 23.5804 | 3.0588 | 11.2123 | 28.0679 | 22.9347 | 51.0026 | 22.4675 | 20.3093 | 30.2862 | 0.4497 | 6.6155 | 28.0551 | 96.9*** |
| 61 | PH_AL2021 | 61.68 | 15.23 | 41.27 | 2.21 | 8.12 | 14.71 | 19 | 33.71 | 9.29 | 10.56 | 14.07 | 0.56 | 6.74 | 16.34 | 71.71*** |
| 62 | PH_AL2022 | 57 | 21.17 | 40.4584 | 2.2511 | 8.2517 | 15.2025 | 11.8256 | 27.0281 | 9.6372 | 8.4997 | 12.8499 | 0.4375 | 4.6858 | 11.5818 | 50.99*** |
| 63 | PH_AN2021 | 73.5 | 18.97 | 44.34 | 2.33 | 8.53 | 16.23 | 61.53 | 77.76 | 9.09 | 17.69 | 19.89 | 0.79 | 14.37 | 32.42 | 200.81*** |
| 64 | PH_AN2022 | 80.91 | 17.56 | 43.0271 | 3.4878 | 12.785 | 36.4941 | 54.89 | 91.3841 | 14.0401 | 17.2189 | 22.2174 | 0.6007 | 11.8284 | 27.4906 | 201.16*** |
| 65 | PH_DeL2021 | 57 | 17 | 37.55 | 2.79 | 10.24 | 23.43 | 55.82 | 79.25 | 12.89 | 19.9 | 23.71 | 0.7 | 12.92 | 34.4 | 190.88*** |
| 66 | PH_DeL2022 | 57 | 17 | 38.9286 | 3.484 | 12.7711 | 36.4148 | 22.1395 | 58.5543 | 15.5014 | 12.0869 | 19.6567 | 0.3781 | 5.9601 | 15.3103 | 102.83*** |
| 67 | PH_DeN2021 | 84 | 31 | 62.78 | 1.48 | 5.42 | 6.56 | 83.8 | 90.36 | 4.08 | 14.58 | 15.14 | 0.93 | 18.16 | 28.93 | 257.96*** |
| 68 | PH_DeN2022 | 84 | 20 | 57.4372 | 4.3463 | 15.9318 | 56.6698 | 39.9242 | 96.594 | 13.1064 | 11.0008 | 17.1112 | 0.4133 | 8.3681 | 14.5691 | 176.4*** |
| 69 | PH_DL2021 | 57 | 16.87 | 39.17 | 2.74 | 10.04 | 22.51 | 13.62 | 36.13 | 12.11 | 9.42 | 15.35 | 0.38 | 4.67 | 11.92 | 63.94*** |
| 70 | PH_DL2022 | 57 | 13 | 37.2116 | 3.5452 | 12.9955 | 37.7058 | 26.5771 | 64.2829 | 16.5016 | 13.854 | 21.5462 | 0.4134 | 6.8285 | 18.3505 | 117.44*** |
| 71 | PH_DN2021 | 76.46 | 17.56 | 41.72 | 3.06 | 11.21 | 28.06 | 60.93 | 88.98 | 12.7 | 18.71 | 22.61 | 0.68 | 13.31 | 31.89 | 210.83*** |
| 72 | PH_DN2022 | 84 | 18.97 | 49.896 | 4.3108 | 15.8017 | 55.7484 | 29.4069 | 85.1553 | 14.9641 | 10.8682 | 18.4944 | 0.3453 | 6.5646 | 13.1566 | 144*** |
| 73 | PY_AL2021 | 471.5 | 10.76 | 93.45 | 16.35 | 59.94 | 802.06 | 2173.44 | 2975.5 | 30.31 | 49.89 | 58.37 | 0.73 | 82.08 | 87.83 | 7322*** |
| 74 | PY_AL2022 | 488.77 | 2.5 | 93.3977 | 23.237 | 85.1781 | 1619.868 | 1919.467 | 3539.335 | 43.0927 | 46.9088 | 63.6978 | 0.5423 | 66.4641 | 71.1624 | 7378*** |
| 75 | PY_AN2021 | 648.69 | 44.82 | 211.76 | 29.89 | 109.56 | 2679.82 | 5492.78 | 8172.59 | 24.45 | 35 | 42.69 | 0.67 | 125.16 | 59.11 | 19158*** |
| 76 | PY_AN2022 | 913 | 1 | 216.0865 | 55.7185 | 204.2433 | 9313.651 | 4476.995 | 13790.65 | 44.6614 | 30.9646 | 54.3456 | 0.3246 | 78.5347 | 36.3441 | 22745*** |
| 77 | PY_DeL2021 | 330.53 | 12 | 147.72 | 24.16 | 88.58 | 1751.69 | 5525.68 | 7277.37 | 28.33 | 50.32 | 57.75 | 0.76 | 133.43 | 90.33 | 18329*** |
| 78 | PY_DeL2022 | 330.53 | 10.5 | 132.4086 | 32.637 | 119.6351 | 3195.519 | 2604.483 | 5800.002 | 42.6928 | 38.5429 | 57.5172 | 0.449 | 70.449 | 53.2057 | 11009*** |
| 79 | PY_DeN2021 | 435 | 12 | 197.01 | 23.1 | 84.68 | 1600.98 | 4107.2 | 5708.18 | 20.31 | 32.53 | 38.35 | 0.72 | 111.99 | 56.84 | 13923*** |
| 80 | PY_DeN2022 | 933 | 12 | 219.2584 | 57.7299 | 211.6165 | 9998.229 | 1998.359 | 11996.59 | 45.6043 | 20.3883 | 49.9543 | 0.1666 | 37.5848 | 17.1418 | 15993*** |
| 81 | PY_DL2021 | 496.77 | 11.43 | 101.74 | 20.08 | 73.62 | 1210.07 | 2463.56 | 3673.63 | 34.19 | 48.78 | 59.57 | 0.67 | 83.73 | 82.3 | 8601*** |
| 82 | PY_DL2022 | 362.2 | 6.76 | 99.7202 | 32.9482 | 120.7758 | 3256.749 | 845.7192 | 4102.468 | 57.2281 | 29.1629 | 64.2302 | 0.2061 | 27.2001 | 27.2764 | 5794*** |
| 83 | PY_DN2021 | 652.74 | 45.09 | 217.34 | 35.3 | 129.41 | 3739.18 | 4933.7 | 8672.88 | 28.13 | 32.32 | 42.85 | 0.57 | 109.13 | 50.21 | 18540*** |
| 84 | PY_DN2022 | 596.11 | 8 | 187.1715 | 42.5684 | 156.04 | 5436.209 | 1322.524 | 6758.733 | 39.392 | 19.4295 | 43.9231 | 0.1957 | 33.1389 | 17.7051 | 9403.8*** |

Max, Maximum; Min, Minimum; GM, Grand Mean; (SEm), Standard Error of Mean (SEm); (CD) 1%, Critical Difference (CD) 1%; EV, Environmental Variance; GV, Genotypic Variance; PV, Phenotypic Variance; ECV, Environmental Coefficient of Variance; GCV, Genotypic Coefficient of Variance; PCV, Phenotypic Coefficient of Variance; H^2^ , Heritability (Broad Sense); GA, Genetic Advance; GA% Mean, Genetic Advance as percentage of mean; G Mean sq, Genotypic mean square. Df, Degrees of freedom; DTF, Days to flowering (days); DTM, Days to maturity (days); PH, Plant height (cm); HSW, hundred seed weight (g); BY, Biological yield (g); HI, Harvest index (%); PY, plot yield (g).

Table S3. Genotypes belong to Population 1, 2 and admixtures population based on STRUCTURE analysis.

| **S. No.** | **Group 1 (Red)** | **Group2 (Green)** | **Admixtures** |
| --- | --- | --- | --- |
| 1 | 3)IG5843 | 1)IG5839 | 11)IG5850 |
| 2 | 6)IG5845 | 2)IG5842 | 18)IG5856 |
| 3 | 9)IG5848 | 4)IG5844a | 26)IG5864 |
| 4 | 12)IG5851a | 5)IG5844b | 29)IG5867 |
| 5 | 25)IG5863 | 8)IG5847 | 31)IG5869 |
| 6 | 7)IG5846 | 14)IG5852 | 38)IG5877 |
| 7 | 10)IG5849 | 15)IG5853 | 40)IG5879 |
| 8 | 13)IG5851b | 17)IG5855 | 44)IG5883 |
| 9 | 16)IG5854 | 19)IG5857 | 46)IG5885 |
| 10 | 22)IG5860 | 20)IG5858 | 48)IG5888 |
| 11 | 24)IG5862 | 21)IG5859 | 60)IG5900 |
| 12 | 33)IG5871 | 23)IG5861 | 81)IG5992 |
| 13 | 34)IG5872 | 27)IG5865 | 88)IG6003 |
| 14 | 35)IG5874 | 28)IG5866 | 92)ILC10252 |
| 15 | 36)IG5875 | 30)IG5868 | 93)ILC11902 |
| 16 | 52)IG5892 | 32)IG5870 | 100)ILC157 |
| 17 | 56)IG5896 | 37)IG5876 | 101)ILC160 |
| 18 | 59)IG5899 | 39)IG5878 | 107)ILC187 |
| 19 | 62)IG5902 | 41)IG5880 | 108)ILC1906 |
| 20 | 63)IG5903 | 42)IG5881 | 112)ILC211 |
| 21 | 64)IG5904 | 43)IG5882 | 117)ILC2377 |
| 22 | 67)IG5907 | 45)IG5884 | 121)ILC3866 |
| 23 | 68)IG5908 | 47)IG5886 | 134)ILC595 |
| 24 | 74)IG5984 | 49)IG5889 | 139)ILC7274 |
| 25 | 75)IG5985 | 50)IG5890 | 146)ILC8797a |
| 26 | 76)IG5986 | 51)IG5891 |  |
| 27 | 78)IG5988 | 53)IG5893 |  |
| 28 | 79)IG5990 | 54)IG5894 |  |
| 29 | 80)IG5991 | 55)IG5895 |  |
| 30 | 84)IG5999 | 57)IG5897 |  |
| 31 | 85)IG6000 | 58)IG5898 |  |
| 32 | 89)IG6006 | 61)IG5901 |  |
| 33 | 94)ILC11913 | 65)IG5905 |  |
| 34 | 95)ILC12022 | 66)IG5906 |  |
| 35 | 96)ILC1272 | 69)IG5909 |  |
| 36 | 105)ILC182 | 70)IG5978 |  |
| 37 | 109)ILC1932 | 71)IG5980 |  |
| 38 | 111)ILC1946 | 72)IG5982 |  |
| 39 | 122)ILC3887 | 73)IG5983 |  |
| 40 | 123)ILC4432a | 77)IG5987 |  |
| 41 | 129)ILC5595 | 82)IG5993 |  |
| 42 | 131)ILC5911 | 83)IG5997 |  |
| 43 | 140)ILC8632 | 86)IG6001 |  |
| 44 | 141)ILC8666 | 87)IG6002 |  |
| 45 | 142)ILC8730 | 90)ILC0Australia |  |
| 46 | 143)ILC8758 | 91)ILC10070 |  |
| 47 | 145)ILC8796 | 97)ILC1312 |  |
| 48 | 148)ILC9793 | 98)ILC1313 |  |
| 49 | 149)ILC9856 | 99)ILC139 |  |
| 50 | 150)ILCItaly | 102)ILC167 |  |
| 51 | 152)ILCRomania | 103)ILC170 |  |
| 52 |  | 104)ILC180 |  |
| 53 |  | 106)ILC184 |  |
| 54 |  | 110)ILC1934 |  |
| 55 |  | 113)ILC213 |  |
| 56 |  | 114)ILC219 |  |
| 57 |  | 115)ILC228 |  |
| 58 |  | 116)ILC234 |  |
| 59 |  | 118)ILC238 |  |
| 60 |  | 119)ILC239 |  |
| 61 |  | 120)ILC35 |  |
| 62 |  | 124)ILC4432b |  |
| 63 |  | 125)ILC445 |  |
| 64 |  | 126)ILC4455 |  |
| 65 |  | 127)ILC5357 |  |
| 66 |  | 128)ILC5588 |  |
| 67 |  | 130)ILC564 |  |
| 68 |  | 132)ILC5919 |  |
| 69 |  | 133)ILC593 |  |
| 70 |  | 135)ILC6025 |  |
| 71 |  | 136)ILC6062 |  |
| 72 |  | 137)ILC6891 |  |
| 73 |  | 138)ILC7187 |  |
| 74 |  | 144)ILC8777 |  |
| 75 |  | 147)ILC8797b |  |
| 76 |  | 151)ILCMoldova |  |
| 77 |  | 153)ILCRussia |  |

Table S4. Marker trait associations (MTAs) identified across the traits and environments having -log(p) value above 4.0

| 28 | HSW_AN2022 | Ca7:30026017 | 7 | 30026017 | 1.31E-05 | 4.882121 |
| --- | --- | --- | --- | --- | --- | --- |
| 29 | PY_N | Ca1:31369599 | 1 | 31369599 | 1.36E-05 | 4.867286 |
| 30 | BY_AL | Ca3:171579 | 3 | 171579 | 1.38E-05 | 4.86101 |
| 31 | PY_AN | Ca6:10230657 | 6 | 10230657 | 1.41E-05 | 4.85086 |
| 32 | HSW_DN2021 | Ca7:30026017 | 7 | 30026017 | 1.63E-05 | 4.789076 |
| 33 | PY_AN | Ca4:6352125 | 4 | 6352125 | 1.81E-05 | 4.742732 |
| 34 | PY_AN2021 | Ca4:6352125 | 4 | 6352125 | 1.88E-05 | 4.726572 |
| 35 | BY_AN | Ca7:41673233 | 7 | 41673233 | 2.10E-05 | 4.677748 |
| 36 | PY_N | Ca6:10230657 | 6 | 10230657 | 2.19E-05 | 4.659681 |
| 37 | BY_AL2021 | Ca3:23273262 | 3 | 23273262 | 2.37E-05 | 4.625671 |
| 38 | PY_DN2022 | Ca1:31369599 | 1 | 31369599 | 2.72E-05 | 4.56479 |
| 39 | BY_DN2021 | Ca7:41673233 | 7 | 41673233 | 3.11E-05 | 4.507177 |
| 40 | BY_AN2022 | Ca6:10230657 | 6 | 10230657 | 3.18E-05 | 4.498144 |
| 41 | BY_AL2021 | Ca1:11522656 | 1 | 11522656 | 3.18E-05 | 4.49783 |
| 42 | BY_DN2021 | Ca6:10230657 | 6 | 10230657 | 3.28E-05 | 4.483501 |
| 43 | DTM_DeL2022 | Ca6:2543062 | 6 | 2543062 | 3.29E-05 | 4.482732 |
| 44 | DTM_DeL2022 | Ca6:2543096 | 6 | 2543096 | 3.29E-05 | 4.482732 |
| 45 | BY_AN | Ca3:10159944 | 3 | 10159944 | 3.55E-05 | 4.449975 |
| 46 | PY_AL2021 | Ca7:10223046 | 7 | 10223046 | 3.93E-05 | 4.405644 |
| 47 | PY_DN2022 | Ca6:10230657 | 6 | 10230657 | 4.04E-05 | 4.393662 |
| 48 | DTM_AL2022 | Ca1:19055085 | 1 | 19055085 | 4.07E-05 | 4.390164 |
| 49 | HSW_N | Ca2:2311917 | 2 | 2311917 | 4.66E-05 | 4.331689 |
| 50 | HSW_DN2022 | Ca2:2311917 | 2 | 2311917 | 4.78E-05 | 4.320778 |
| 51 | BY_DN2022 | Ca3:10159944 | 3 | 10159944 | 5.20E-05 | 4.283749 |
| 52 | PY_AN2021 | Ca6:10230657 | 6 | 10230657 | 5.43E-05 | 4.264896 |
| 53 | PY_AN2022 | Ca6:10230657 | 6 | 10230657 | 5.61E-05 | 4.250837 |
| 54 | DTM_DN2021 | Ca8:184496 | 8 | 184496 | 5.64E-05 | 4.248722 |
| 55 | DTM_DeL2021 | Ca6:2543062 | 6 | 2543062 | 5.73E-05 | 4.241681 |
| 56 | DTM_DeL2021 | Ca6:2543096 | 6 | 2543096 | 5.73E-05 | 4.241681 |
| 57 | HI_DeL2022 | Ca2:28949909 | 2 | 28949909 | 5.82E-05 | 4.235225 |
| 58 | PY_AN2021 | Ca7:41673233 | 7 | 41673233 | 5.96E-05 | 4.225017 |
| 59 | HI_AL2022 | Ca4:45204033 | 4 | 45204033 | 5.99E-05 | 4.222748 |
| 60 | HSW_AN2021 | Ca2:2311917 | 2 | 2311917 | 6.21E-05 | 4.206587 |
| 61 | HI_AL2021 | Ca7:3796894 | 7 | 3796894 | 6.23E-05 | 4.205585 |
| 62 | HSW_DeN2022 | Ca7:42587321 | 7 | 42587321 | 6.91E-05 | 4.160415 |
| 63 | DTM_AN2022 | Ca8:184496 | 8 | 184496 | 7.14E-05 | 4.146454 |
| 64 | DTM_AL2022 | Ca3:35167359 | 3 | 35167359 | 7.28E-05 | 4.138029 |
| 65 | PY_AN2021 | Ca6:9109096 | 6 | 9109096 | 7.70E-05 | 4.113287 |
| 66 | HI_DN2022 | Ca3:37444451 | 3 | 37444451 | 8.08E-05 | 4.09278 |
| 67 | PY_N | Ca4:6352125 | 4 | 6352125 | 8.14E-05 | 4.089531 |
| 68 | HSW_AN2021 | Ca2:26121495 | 2 | 26121495 | 8.50E-05 | 4.07047 |
| 69 | PY_AN2021 | Ca3:10159944 | 3 | 10159944 | 8.60E-05 | 4.065517 |
| 70 | BY_AL2021 | Ca1:27012660 | 1 | 27012660 | 8.64E-05 | 4.06362 |
| 71 | DTM_AN2022 | Ca8:9151805 | 8 | 9151805 | 8.98E-05 | 4.04674 |
| 72 | BY_AN2022 | Ca7:41673233 | 7 | 41673233 | 9.04E-05 | 4.043597 |
| 73 | PY_AL | Ca7:10223046 | 7 | 10223046 | 9.09E-05 | 4.041445 |
| 74 | PY_AL2022 | Ca4:45204033 | 4 | 45204033 | 9.10E-05 | 4.041015 |
| 75 | HI_DeN2021 | Ca3:37444451 | 3 | 37444451 | 9.38E-05 | 4.027806 |

DTF, Days to flowering (days); DTM, Days to maturity (days); PH, Plant height (cm); HSW, hundred seed weight (g); BY, Biological yield (g); HI, Harvest index (%); PY, plot yield (g).

Table S5: Common MTAs with pleotropic markers effect identified after Bonferroni-corrected p-value (–log10(p) > 4.95)

| SNP | Trait | Chromosome | Position (Mb) | *P*-value | -log10(*p*) |
| --- | --- | --- | --- | --- | --- |
| Ca2:2311917 | HSW_DN2021, | 2 | 2.31 | 9.87E-09 | 8.005556 |
|  | HSW_AN2022, | 2 | 2.31 | 2.70E-08 | 7.568951 |
|  | HSW_AN | 2 | 2.31 | 8.00E-08 | 7.097058 |
| Ca4:8669498 | HSW_DN2021 | 4 | 8.67 | 2.73E-06 | 5.564497 |
|  | HSW_AN2022 | 4 | 8.67 | 2.90E-06 | 5.537057 |
| Ca6:10230657 | BY_N | 6 | 10.23 | 3.59E-07 | 6.444971 |
|  | BY_DN2022 | 6 | 10.23 | 3.83E-06 | 5.417043 |
|  | BY_AN2021 | 6 | 10.23 | 7.72E-06 | 5.112628 |
|  | PY_DN2021 | 6 | 10.23 | 8.65E-06 | 5.062903 |
| Ca7:41673233 | PY_DN2022 | 7 | 41.67 | 1.09E-07 | 6.963979 |
|  | BY_N | 7 | 41.67 | 1.38E-06 | 5.860313 |
|  | BY_DN2022 | 7 | 41.67 | 6.44E-06 | 5.190981 |
| Ca8:10963827 | DTM_DL2021 | 8 | 10.96 | 1.08E-06 | 5.967427 |
|  | DTM_AL2022 | 8 | 10.96 | 1.06E-05 | 4.97535 |

DTF, Days to flowering (days); DTM, Days to maturity (days); PH, Plant height (cm); HSW, hundred seed weight (g); BY, Biological yield (g); HI, Harvest index (%); PY, plot yield (g).

Table S6. Number of accessions performed outstanding and stable for the traits under study

| Trait | Timely | Mean | Late | Mean |
| --- | --- | --- | --- | --- |
| DTF | ILC6891 | 70.48 | IG5984 | 46.28 |
| DTM | IG5861 | 125.05 | IG5877 | 86.93 |
| PH | IG5902 | 69.07 | IG5983 | 46.51 |
| HSW | IG5984 | 31.88 | IG5984 | 31.05 |
| BY | IG5901 | 1203.33 | ILC8666 | 505.25 |
| HI | ILC6062 | 37.30 | IG5839 | 36.26 |
| PY | IG5866 | 281.42 | ILC8666 | 201.01 |
